# Mental health of people detained within the justice system in Africa: systematic review and meta-analysis

**DOI:** 10.1186/s13033-019-0273-z

**Published:** 2019-05-06

**Authors:** Aish Lovett, Hye Rim Kwon, Khameer Kidia, Debra Machando, Megan Crooks, Gregory Fricchione, Graham Thornicroft, Helen E. Jack

**Affiliations:** 1 Harvard College, 28 Fernald Drive, Cambridge, MA 02138 USA; 2 Institute of Psychology, Psychiatry, and Neuroscience, King’s College London, 16 De Crespigny Park, Camberwell, London, SE5 8AB UK; 3 Kushinga, 8 Collina Close, Borrowdale, Harare, Zimbabwe; 4 Brigham and Women’s Hospital, 75 Francis Street, Boston, MA 02115 USA; 5 Department of Psychiatry, University of Zimbabwe, 630 Churchill Avenue, Harare, Zimbabwe; 6 The Ulster Hospital, Upper Newtownards Road, Dundonald, Belfast, BT16 1RH UK; 7 Department of Psychiatry, Massachusetts General Hospital, 55 Fruit Street, Boston, MA 02114 USA; 8 Centre for Global Mental Health, Institute of Psychology, Psychiatry, and Neuroscience, King’s College London, London, UK; 9 Department of Medicine, University of Washington, 1959 NE Pacific Street, Seattle, WA 98195 USA

## Abstract

Worldwide, people with mental disorders are detained within the justice system at higher rates than the general population and often suffer human rights abuses. This review sought to understand the state of knowledge on the mental health of people detained in the justice system in Africa, including epidemiology, conditions of detention, and interventions. We included all primary research studies examining mental disorders or mental health policy related to detention within the justice system in Africa. 80 met inclusion criteria. 67% were prevalence studies and meta-analysis of these studies revealed pooled prevalence as follows: substance use 38% (95% CI 26–50%), mood disorders 22% (95% CI 16–28%), and psychotic disorders 33% (95% CI 28–37%). There were only three studies of interventions. Studies examined prisons (46%), forensic hospital settings (37%), youth institutions (13%), or the health system (4%). In 36% of studies, the majority of participants had not been convicted of a crime. Given the high heterogeneity in subpopulations identified in this review, future research should examine context and population-specific interventions for people with mental disorders.

**Electronic supplementary material:**

The online version of this article (10.1186/s13033-019-0273-z) contains supplementary material, which is available to authorized users.

## Introduction

Investments in mental health care and research are critical: globally, psychiatric and neurological disorders comprise approximately 13% of the disability-adjusted life years and nearly one-third of years lived with disability [1]. The prevalence of mental illness is higher among people detained within the justice system (PDJS) compared to the general population [2–9]. In this review, PDJS are defined as people detained or incarcerated in prisons, jails, youth institutions, or forensic inpatient units of hospitals. In accordance with the World Health Organization (WHO)’s definition, a forensic inpatient unit is “exclusively maintained for the evaluation or treatment of people with mental disorders who are involved with the justice system. These units can be located in mental hospitals, general hospitals, or elsewhere.” [10]. This review uses an inclusive definition of detention to respond to the scarcity of research on PDJS’s mental health in Africa, particularly for people detained outside of prisons. International systematic reviews on the mental health of PDJS show that populations in prisons are multiple times more likely to have several major mental disorders [2, 4, 5, 9] and have a three to sixfold higher risk of death by suicide [3]. More than 10 million people are held in penal institutions worldwide [11], and have increased risk of adverse outcomes such as all-cause mortality, suicide, self-harm, violence, and victimization [3]. The prevalence of mental disorders in African countries is of particular concern due to resource constraints for mental health and expansion of the movement to shift resources from institutions to community-based care in low- and middle-income countries (LMICs) [12, 13]. In 2017, there were 2.5 total mental health beds per 100,000 population in African countries as a whole, 80% of which were in psychiatric hospitals [10], illustrating a contrast between where most existing mental health resources go (hospitals) and where services may be needed and, in reality, delivered (the community). There is, however, an international movement, including in Africa, to shift resources from a country’s psychiatric hospital or hospitals to other forms of mental health services [14, 15]. This movement recognizes that most people in low-resource settings have been receiving care in the community (if at all). However, in contexts with inadequate community health care, people previously in institutions may face increased risk of diversion to the justice system [15–18]. As health resources shift to better support mental health care in primary care and outpatient services in some African countries, research is needed on mental health interventions for justice-involved populations with elevated prevalence rates.

However, there has been minimal attention to the mental health of PDJS in international data collection and guidelines [19]. We surveyed United Nations (UN) and WHO guidelines on mental health and detained populations from the past 15 years (Table 1) and found that the mental health of PDJS has not been present in the majority of publications. The exclusion of the justice system from research or policy priorities contrasts to consensus in international prison literature which explicitly requires extensive mental health care services (see Table 1).

**Table 1:** International guidelines on mental health and people detained within the justice system

Document title, year of publication	Purpose	How does the document discuss mental health and detention?
WHO mental health action plan 2013–2020 (2013) [72]	An action plan for Member States which outlines ways to promote mental health, prevent mental disorders, protect human rights of persons affected by mental health conditions and to reduce mortality, morbidity and disability for people with mental disorders	Mental health: Large focus on four objectives: strengthening leadership and governance for mental health; expanding service coverage for mental disorders; implementing strategies for promotion and prevention in mental health, and strengthening the evidence base for mental health research
		Detention: Mentions that inappropriate detention is more common for people with mental disorders and encourages collaboration with judicial sectors in all four objectives. Little is said about addressing the mental health of those that are specifically detained
Time to deliver, report of the who independent high-level commission on noncommunicable diseases (2018) [73]	Report aims to facilitate the implementation of Sustainable Development Goal 3.4, ‘reducing premature mortality from NCDs’, as progress so far has been inadequate	Mental health: Recognizes the global impact of mental disorders and specifically discusses mental health in each of its recommendations
		Detention: No mention of people detained within the justice system
World Health Organization assessment instrument for mental health systems (AIMS 2.2) (2005) [74]	Document provides guidance on data collection for WHO AIMS 2.2, a tool for collecting information on key components of a mental health system	Mental health: Assessment tool assesses the following: policy and legislation, mental health services, mental health in primary health care, human resources, public education and links with other sectors, and monitoring and research
		Detention: Includes the assessment of prison mental health services and forensic inpatient units
WHO mental health and development: targeting people with mental health conditions as a vulnerable group (2010) [75]	Report presents evidence showing that people with mental health conditions comprise a vulnerable group and provides recommendations for the implementation of policies that aim to protect this marginalized group	Mental health: Highlights the need for development programs to pay more attention to people with mental health conditions as they are among the most marginalized and vulnerable groups in society but are often overlooked
		Detention: Recognizes that there is a significant problem to be addressed—people with mental health conditions are directed towards prisons, where they often do not have access to adequate mental health provisions and services
WHO checklist for evaluating a mental health policy (2005) [76]	A checklist for evaluating mental health policies	Mental health: Evaluates the process of policy development and the policy’s contents. Emphasizes a multisectoral, human-rights approach to developing policies
		Detention: Suggests consulting with the justice system when developing policies but otherwise does not consider the mental health of detained people
UN General Assembly, Report of the United Nations High Commissioner for human rights: mental health and human rights (2017) [77]	To identify some of the main challenges faced by people with mental health conditions or psychosocial disabilities and recommends policies which would support the full realization of human rights of this population	Mental health: Emphasizes that human rights of persons with mental illnesses are vastly neglected in society. It stresses the importance of changing policies and law to protect the human rights of this vulnerable population
		Detention: No mention of people detained within the justice system
UNOPS technical guidance on prison planning (2016) [78]	A guide to prison infrastructure development based on a human rights approach	Mental health: Recognizes that people detained in prisons with mental health conditions constitute a vulnerable group that may require separate accommodation
		Health in prisons: Recognizes that there is a lack of practical guidance on prison infrastructure development which takes into consideration the Standard Minimum Rules for the Treatment of Prisoners
UNODC handbook on prisoners with special needs (2009) [79]	Outlines the special needs of eight groups of adults in prisons which have a particularly vulnerable status and provides recommendations for policymakers	Mental health: Thoroughly describes the needs of people in prisons with mental health conditions and recognizes them as a vulnerable group. Highlights that promotion of mental well-being should be a key element of prison management and policies
		Health in prisons: Recognizes that imprisonment is a disproportionately harsh punishment for many people in vulnerable groups. Suggests that their special needs are better addressed away from prisons, as the harsh prison environment would likely exacerbate any existing problems
United Nations expert group meeting on mental well-being, disability and disaster risk reduction (2014) [80]	Provides guidelines for countries’ Disaster Risk Reduction (DRR) policies so that they include mental health and disability as a priority	Mental health: States the need to include mental well-being *and* mental disabilities in all DRR frameworks, as it optimizes resilience to disasters
		Detention: No mention of people detained within the justice system
United Nations standard minimum rules for the treatment of prisoners (the Nelson Mandela Rules) (2015) [81]	Revised version of rules that set out the minimum standards for the treatment of people detained in prisons	Mental health: Highlights that all individuals with mental conditions in prisons should have access to the same care they would have in the community and should be transferred to a hospital if required
		Health in prisons: Reaffirms that the punishment caused by imprisonment is by depriving individuals of liberty, so prison systems should not aggravate their suffering further

Despite the burden of mental disorders among PDJS in Africa, research in this area is sparse [3, 19]. Existing international systematic reviews on the mental health of PDJS have either included studies in only one African country, Nigeria [2], or none at all [4, 5, 20, 21]. Moreover, these reviews do not report methodological bias or ethics procedures data of included studies, and the search criteria of most do not include institutions that detain justice-involved youth or inpatients in forensic psychiatry units [2, 4, 5, 21], even though this is where many people with mental illness may be detained and are similar to prisons in some countries [22]. A recent systematic review on the influence of prison climate on mental health resulted in studies from only high-income countries [21]. Similarly, a review of psychological therapies for PDJS internationally did not result in studies from LMIC countries other than India, Iran, and China [20]. Importantly, however, its search criteria included youth and participants in secure hospitals, while the other systematic reviews include only those in prisons and jails [2, 4, 5, 21].

Given the paucity of research focused on Africa and the resource constraints of these countries, this review aims to understand the scope of knowledge on the mental health of PDJS in Africa and to identify gaps in the literature, in order to inform future research, interventions, harm reduction, efforts, and policy. Given our study aims, we did not limit our search and selection to a single study design or outcome measure. In contrast to previous reviews investigating only prevalence, specific study types, or restricted to prisons, the broad scope of this review responds to the scarcity of research in African contexts and the systems-wide nature of detention and issues surrounding the mental health of PDJS.

## Methods

The review followed guidelines of the Preferred Reporting Items for Systematic Review and Meta-analyses (PRISMA) [23], and the meta-analysis of observational studies in epidemiology (MOOSE) [24], both of which are found in Additional file 1: Appendices S1 and S2. The protocol was registered in the International Prospective Register of Systematic Reviews (PROSPERO). The Registration Number is CRD42018098852.

### Search strategy and selection criteria

We searched PubMed, PsycINFO, Embase, Web of Science, and Africa Index Medicus for studies dating from the inception of the database to 16 November 2017, and published in English or French, major research languages on the African continent. Because the inception of databases are constantly updated as new articles are digitized, searches were run without date limits. These databases represent major features of this paper: international health databases (PubMed, Embase, Web of Science), a psychiatry-specific database (PsycINFO), and an Africa-specific database (African Index Medicus). Africa Index Medicus is managed by the WHO, and is one of two major African health databases. The other is African Journals Online, which is not health specific. Our search strategy included terms related to the following ideas: (1) Africa (defined as the WHO Africa Region [25]), (2) detention or incarceration, and (3) mental health. Search syntax and controlled vocabulary words were modified for each database. Complete search terms for each database are in Additional file 1: Appendix S3.

Only primary research studies published in peer reviewed journals in English or French that met the following PICOS criteria were included. Participants included any people detained by the state, regardless of whether they had been sentenced to a crime. We excluded people formerly detained if data was collected after their release, and excluded people detained for immigration (similar to prior research on detained populations) [3]. We included studies that related to a mental illness as defined according to the DSM-V, but included studies that used other diagnostic criteria. Due to the scarcity of systematic research in this area, we included both qualitative and quantitative studies, including systematically collected data on policy, health systems, and conditions of detention, such as availability of physical or human resources, services, and the legal process. We included studies in the WHO Africa Region [25] and in any setting in which people were involuntarily held by the government for being accused or convicted of committing a crime. We excluded the settings of house arrest or non-forensic psychiatric hospitals in which people are involuntarily committed for disease alone.

### Data collection

**Fig. 1:**
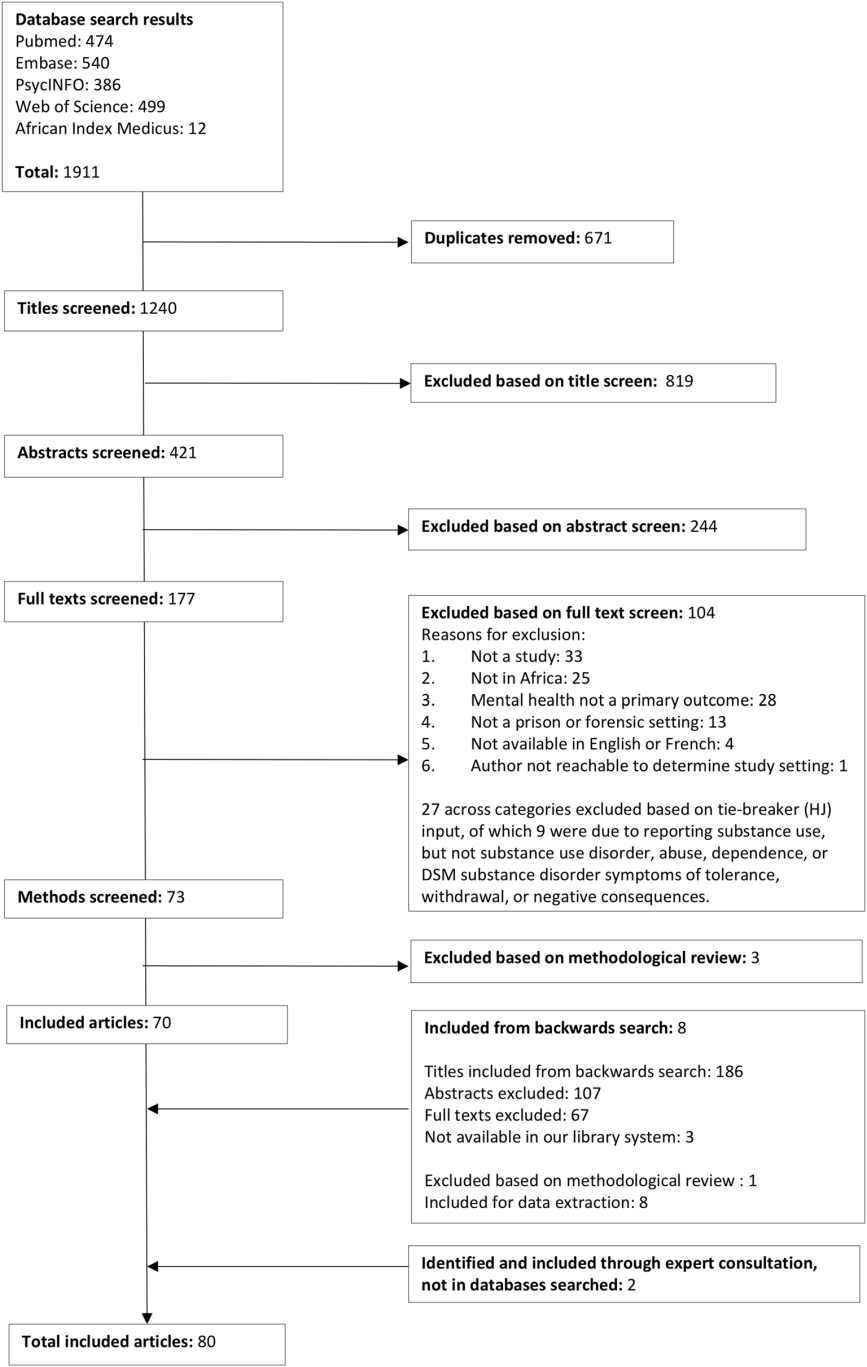
Flowchart

After the search was conducted and duplicates removed, two researchers (AL, HK—trained undergraduate students) separately screened the titles, abstracts, and full texts of studies to assess whether they met the inclusion criteria, reconciling differences between each step through discussion. A third reviewer (HJ) acted as a tie breaker if the other screeners could not come to consensus. Following the full text screen, a backward search was conducted on all articles selected for inclusion. The results of the backward search were screened for inclusion following the same protocol as the initial search results (Fig. 1). If a paper identified could not be located using multiple university library systems, we attempted to contact the study author. If the author could not be contacted, we excluded this paper as it was not possible to access the full text. We did not contact authors to obtain missing data or details on methods.

### Risk of bias assessment

Risk of bias was assessed using the following separate tools for each study type. Pre-post studies: Quality assessment tool for quantitative studies [26]; RCTs: Cochrane Collaboration’s tool for assessing risk of bias [27]; qualitative: Critical Appraisal Skills Program (CASP) Checklist for Qualitative Studies [28]; prevalence: Prevalence Critical Appraisal Checklist [29]. We did not assess risk of bias for validation studies or structured reviews of health systems. The small number of these studies included in the review and the methodologic heterogeneity of structured reviews or descriptions of health systems make them difficult to compare with a single tool. We did not report a single overall risk of bias score for each study, as these scores typically involve arbitrarily weighting different domains of risk of bias [30]. Rather, we reported grouped studies into categories of high, medium, and low risk of bias based on the overall assessment generated by the methods assessment instruments. The methodological assessment was used to exclude studies that had either (1) insufficient information on methods or (2) a clear methodological flaw or inconsistency.

### Data extraction

Data on study characteristics, sample characteristics, and study outcomes were extracted from each study (Tables 2, 3). Systematic reviews have the potential to elevate studies with poor ethics standards [31]; therefore, we extracted data on the presence or absence of documentation of an ethics committee review and informed consent procedures. If institution conditions or the laws or policy of the health or justice system were described, we extracted these descriptions and categorized the data into common findings (Additional file 1: Appendix S9).

**Table 2:** Prevalence studies

Reference *If same sample as another study in list	Study design	Study setting	Country setting	Comparison [If yes (Y), describe; no (N)]	Strategy, whether sample size calculation was reported for non-census strategies	Ethics reporting (documented ethics committee approval; described informed consent procedure)	Participants characteristics (sample size, mean age, percent male) *Indicates gender as inclusion criteria	Inclusion criteria (excluding age criteria)	Trial status category (C = over 50% convicted; NC = over 50% not convicted; NC/A = over 50% “awaiting trial”; JI = over 50% youth justice-involved; U = unclear; NA = not applicable; NS = not stated)	Assessment instruments (diagnostic or screening tool)	Primary outcomes (p-value listed if provided in study)	Methods risk of bias score
Abdulmalik et al. 2014 [82]	Prevalence	Prison	Nigeria	N	Census	Yes, Yes	725, 31.1, 98.7%	Awaiting trial and remanded; GHQ-12 ≥ 5 for phase 2	NC/A *Awaiting trial	GHQ-12 (S), MINI (D)	56.6% prevalence of mental illness (MINI), assessed after scoring ≥ 5 GHQ-12. Depression 20.8%; alcohol dependence 20.6%; substance dependence 20.1%; suicidality 19.8%; antisocial personality disorder 18%; panic disorder 8.3%; OCD 8.3%; PTSD 3.3%; GAD 2.8%; psychosis 1.1%	Low
Agbahowe et al. 1998 [83]	Prevalence	Prison	Nigeria	N	Census	No, No	100, 31.4, 93%	Convicted; GHQ-30 > 4 for phase 2	C *Convicted and no other classification (81%), convicted but detained (6%); convicted and condemned to death (13%)	GHQ-30 (S), Psychiatric Assessment Schedule (PAS) (D), SCAN (D)	34% ≥ 4 score on GHQ-30; 100% of GHQ-30 ≥ 4 cases had DSM IIIR Axis I diagnosis	Low
Agboola et al. 2017 [84]	Prevalence	Prison	Nigeria	N	Random, N	Yes, Yes	94, 28.5, 100%*	Male	NS, awaiting trial and convicted	GHQ-28 (S), Present State Examination (PSE) (D), PULSES (S)	39% prevalence of psychiatric morbidity (PSE). As measured by PSE, 20.2% of total participants diagnosed with depression; 14.8% anxiety; 3.2% schizophrenia; 1.1% mania; 1.1% OCD. 57.4% participants scored ≥ 5 on the GHQ-28. Of participants with psychiatric diagnosis, 39.7% with co-morbid physical illness (PULSES)	Low
Akkinawo 1993 [85]	Prevalence	Prison	Nigeria	N	Random, NS	No, No	136, NS, 93.4%	NA	NS	API (S), BDI (S)	20.86% depression (BDI); 35.29% general mood disorder; 30.15% general psychopathology; 26.47% sleep disorder (API)	Medium
*Armiya’u et al. 2013 “Prevalence of…” [86]	Prevalence	Prison	Nigeria	N	NS	No, No	608, 32.1, 100%*	Males (though unclear); NA for phase 1, > 4 GHQ-28 for phase 2	NC/A *60% awaiting trial, 40% convicted	GHQ-28 (S), CIDI (D)	57% psychiatric morbidity (CIDI), administered to those with GHQ-28 score ≥ 4	Medium
*Armiya’u et al. 2013 “A study of…” [87]	Prevalence	Prison	Nigeria	N	NS	Yes, No	608, 32.1, 100%*	Males (though unclear); NA for phase 1, > 4 GHQ-28 for phase 2	NC/A *60% awaiting trial, 40% convicted	GHQ-28 (S), PULSES (S), CIDI (D)	57% psychiatric morbidity (CIDI), administered to those with GHQ-28 score ≥ 4. 18% prevalence of co-morbid physical illness (comorbid illness indicated by PULSES)	Medium
*Beyen et al. 2017 [88]	Prevalence	Prison	Ethiopia	N	Random, Y	Yes, Yes	649, 27.8, 89.8%	NA	NS	GAD-7 (S), K10 (S), PHQ-9, (S) OSS (S), questionnaire (S)	83.4% psychological distress (K10); 43.8% signs of depression (PHQ-9); 36.1% anxiety (GAD-7); 45.1% without social support (OSS). 17% suicidal ideation; 16.6% already planned to commit suicide; 11.9% at least one suicide attempt while in prison (questionnaire)	Low
*Dachew et al. 2015 [89] (same sample as Beyen)	Prevalence	Prison	Ethiopia	N	Random, Y	Yes, Yes	649, 27.8, 89.8%	NA	NS	K10 (S), questionnaire (S), MSPSS (S)	83.4% psychological distress (K10). 43.6% of the respondents feel that they had been discriminated by their families, friends and significant others because of their imprisonment (questionnaire or MPSS, source not stated). 64.7% “yes” reported social support; 35.3 “no” (MPSS)	Low
*Dadi et al. 2016 [90] (same sample as Beyen)	Prevalence	Prison	Ethiopia	N	Random, Y	Yes, Yes	649, 27.8, 89.8%	NA	NS	GAD-7 (S)	36.1% anxiety (GAD-7)	Low
Fatoye et al. 2006 [91]	Prevalence	Prison	Nigeria	N	Census	No, Yes	303, 31.2, 96.4%	NA	NC/A *81.3% awaiting trial, 18.7% sentenced	GHQ-30 (S), HADS (S)	87.8% possible psychiatric morbidity (GHQ-30 ≥ 5). 85.3% HADS ≥ 8 significant depressive symptoms	Low
Ibrahim et al. 2015 [92]	Prevalence	Prison	Ghana	N	Random and census, NS	Yes, Yes	100, 37, 89%	NA	NS	K10 (S)	64% K10 scores ≥ 25 indicating moderate to severe mental distress	Low
Kanyanya 2007 [93]	Prevalence	Prison	Kenya	N	Census	No, Yes	76, 33.5, 100%*	Males, convicted of sex offense	C	SCID (D), IPDE (D)	35.5% DSM-IV Axis 1 disorder (SCID). 34% prevalence of DSM-IV Axis 2 disorders (SCID and IPDE)	Medium
*Mafullul 2000 [94]	Prevalence	Prison	Nigeria	N	Census	No, No	118, 33.9, 96%	Convicted of homicide	C	Psychiatric record (D)	Psychotic disorders and substance use disorders, including alcohol intoxication, suggested to be held to accountable for 39.8% persons’ offenses. 45% of participants had positive histories of substance use disorders	High
*Mafullul et al. 2001 [95]	Prevalence	Prison	Nigeria	N	Census	Yes, No	118, 33.9, 96%	Convicted of homicide	C	Psychiatric record (D)	68% of the accused referred for pre-trial psychiatric assessment had killed victims as a result of psychotic motives. Court recognized that alcohol intoxication and psychotic motives accounted for the offenses of 24% of the accused. Study indicates that substance use disorders may have accounted for offenses of 45% of accused	High
Majekodunmi et al. 2017 [96]	Prevalence	Prison	Nigeria	N	Random, Y	Yes, Yes	196, 32.8, 100%*	Male, those with no past treatment for mental illness, no debilitating physical illness	NC/A, 69.4% awaiting trial, 30.6% convicted	SCID-IV (D), Montgomery–Asberg Depression Rating Scale (MADRS) (S),, Medical history questionnaire (S)	30.1% depression; mean total MADRS score 23.9 among awaiting trial participants. 35.0% depression; mean total MADRS score 25.5 among awaiting trial participants. From medical history questionnaire, resence of physical complaints (p = 0.014) and chronic illness (p = 0.023) associated with depression among awaiting trial participants; family history of psychiatric illness associated with depression among convicted participants (p = 0.046)	Low
Mela et al. 2014 [97]	Prevalence	Prison	Ethiopia	N	Census	Yes, Yes	546, NS, 94.3%	Convicted of homicide	C	SRQ-20 (S) SCID-IV (D)	35.5% SRQ-indicated psychological distress. Among 316 participants who agreed to undergo a psychiatric interview for Axis I diagnosis (SCID-IV), 41.8% history of substance use disorder; 25% depression; 10.1% adjustment disorder; 7.6% anxiety disorder; 0.6% PTSD; 0.6% psychotic disorder; 1.6% psychotic disorder due to medical condition; 15.8% personality disorder (SCID)	Low
Naidoo and Mkize 2012 [98]	Prevalence	Prison	South Africa	N	Random, Y	Yes, Yes	193, 30.5, 95.8%	NA	C *62% convicted, 38% awaiting trial	MINI (D)	55.4% Axis 1 disorder from MINI	Medium
Nseluke and Siziya 2011 [99]	Prevalence	Prison	Zambia	N	Random, Y	Yes, Yes	206, 33.7, 83%	NA	NC/A *74.3% awaiting trial, 23.3% sentenced, 1.9% probation violation, 0.5% parole violation	SRQ (S)	63.1% mental illness as indicated by SRQ	Low
Osasona and Koleoso 2015 [100]	Prevalence	Prison	Nigeria	N	Random and census, NS	Yes, Yes	252, 33.7, 90.9%	NA	C *57.1% sentenced, 42.9% awaiting trial	SRQ-20 (S), HADS (S)	84.5% of the respondents had at least one type of psychiatric morbidity (SRQ and HADS combined). Prevalence of general psychiatric morbidity, SRQ-20 score ≥ 5, 80.6%. 72.6% and 77.8% were found to be positive for depression and anxiety symptoms respectively on the HADS	Low
Schaal et al. 2012 [101]	Prevalence	Prison	Rwanda	Y (genocide survivors)	Random, NS	Yes, Yes	269, 48.5, 65.8% (genocide perpetrators); 114, 46.6, 36.3% (survivors)	Perpetrators of the Rwandan genocide, over 18 years in 1994	C *89.6% convicted, 10.4% not sentenced	PTSD Symptom Scale-Interview (PSS-I) (D), PDS Event Scale (S), Hopkins Symptom Checklist-25 (HSCL-25) (S), suicidality scale from the MINI (S)	Diagnostic criteria for PTSD met by 13.5% perpetrators and 46.4% of interviewed survivors (p < 0.001) (PSS-I). Clinically significant anxiety prevalence 35.8% among perpetrators (HSCL-25); 58.9% among survivors (p < .001). Depression in both groups (46% survivors vs. 41% perpetrators) (HSCL-25). 18.6% perpetrators and 19.3% survivors had suicide risk (MINI). Perpetrators with more severe depression symptoms (HSCL-25) reported high levels of trauma confrontation (PDS) and had not participated in killings	Low
*Uche and Princewill 2015 “Clinical factors…” [102]	Prevalence	Prison	Nigeria	N	Random, Y	Yes, Yes	400, 33.8, 98%	Awaiting trial; BDI-screen positive for phase 2	NC/A* awaiting trial	BDI (S), SCAN Depression Component (D)	42% BDI > 10 screen fulfilling the criteria for current depressive disorder. 42% fulfilled SCAN criteria for current depression disorder diagnosis	Low
*Uche and Princewill 2015 “Prevalence…” [103]	Prevalence	Prison	Nigeria	N	Random, Y	Yes, Yes	400, 33.8, 98%	Awaiting trial; BDI-screen positive for phase 2	NC/A *89% awaiting trial, 5% convicted, 0.1% assigned legal category of “lunatics,” death row condemned 5%, serving life imprisonment jail terms 0.5%	BDI (S), SCAN depression component (D)	42% BDI > 10 screen fulfilling the criteria for current depressive disorder. 42% fulfilled SCAN criteria for current depression disorder diagnosis	Low
Barrett et al. 2007 [52]	Prevalence	Forensic ward	South Africa	N	Census	Yes, No	71, NS, 94.4%	Psychiatric referrals	NC *Detained “state patients” accused but found unfit to stand trial or not responsible, referred to forensic ward	Psychiatric record (D)	Schizophrenia (35.2%), mental retardation (22.5%) and psychoses other than schizophrenia (11.3%) most prevalent, followed by bipolar disorder (5.6%). 84.5% not able to stand trial and not accountable; 7% not fit to stand trial and accountable; 8.5% not accountable and fit to stand trial	Medium
Buchan 1976 [104]	Prevalence	Forensic ward	Zimbabwe	N	Census	No, No	256, NS, NS	Psychiatric referrals	U *Referrals to hospital	Psychiatric record (D)	Prevalence of schizophrenia 44%; epilepsy 22%	High
Calitz et al. 2006 [105]	Prevalence	Forensic ward	South Africa	N	Census	Yes, No	514, 30 (median), 94.6%	Psychiatric referrals	NC/A *Awaiting trial, referrals to hospital	Psychiatric record (D)	46% psychiatric prevalence.	Medium
du Plessis et al. 2017 [106]	Prevalence	Forensic ward	South Africa	N	Census	Yes, No	505, NA, 94%	Awaiting trial; psychiatric referrals	NC/A *Awaiting trial, referrals to hospital	Psychiatric record (D)	Those not accountable significantly more likely to have mental illness (p = 0.0001) and be diagnosed with schizophrenia (p = 0.0001), intellectual disability (p = 0.0001), and substance-induced psychotic disorder (p = 0.02) than those not accountable. 98% of those found not accountable had mental illness. 66% total sample had known history of substance abuse	Low
Hayward et al. 2010 [107]	Prevalence	Forensic ward	Malawi	N	Census	No, No	283, 30.4, 91.5%	Psychiatric referrals	U *Detained in hospital	Psychiatric record (D)	Prevalence of schizophrenia 35.5%; substance misuse 32.5%; 19.8% alcohol and 23% illicit substance; depression 3%; mania or personality disorder 0%; epilepsy 8.1%	Medium
Hemphill and Fisher 1980 [108]	Prevalence	Forensic ward	South Africa	N	Census	No, No	604, NS, 100%*	Males (though unclear); psychiatric referrals	NC *Pre-trial referrals to hospital	Psychiatric record (D)	52% substance abuse of drugs, alcohol, or both. Prevalence of psychosis (53%), severe psychopathy without psychosis (21%), and non-psychotic conditions including neurosis, mild personality disorder, eplepsy and mental retardation (26%). More than 70% of patients with psychopathy screened positive for substance abuse of alcohol, drugs or both	High
Khoele et al. 2016 [109]	Prevalence	Forensic ward	South Africa	N	Census	Yes, No	32, 29.8, 0%	Women; charged with murder or attempted murder, psychiatric referrals	NC *Pre-trial referrals to hospital	Psychiatric record (D)	59% psychiatric diagnosis; 28% psychotic; 25% mood disorders; 6% substance disorders; 19% attempted suicide	Medium
Marais and Subramaney 2015 [53]	Prevalence	Forensic ward	South Africa	N	Census	Yes, Yes	114, 32, 87%	Psychiatric referrals	NC *Detained “state patients” accused but found unfit to stand trial or not responsible, referred to forensic ward	Psychiatric record (D)	Past psychiatric history (59%); substance abuse history (71%). 69% psychotic disorders; 44% schizophrenia. Bipolar mania 4%; major depressive disorder 4%; epilepsy 4%. Alcohol the most frequently abused substance (57%); cannabis 47%. 37% reported a history of polysubstance abuse	Medium
Matete 1991 [110]	Prevalence	Forensic ward	Kenya	N	Census	No, No	51, 28.8, 90.2%	Psychiatric referrals	NC *Detained in hospital: court referrals to hospital, referred to as “criminal remands”	Psychiatric record (D)	86.3% mental illness	Medium
Mbassa 2009 [111]	Prevalence	Forensic ward	Cameroon	N	Random, NS	No, No	12, 18.3, 66.7%	Convicted of homicide	C *Convicted, detained in hospital	Psychiatric record, ICD-10 criteria (D)	41.7% schizophrenia; delirium 25%; personality disorder 8.3%	High
Menezes 2010 [112]	Prevalence	Forensic ward	Zimbabwe	N	Census	Yes, Yes	39, 35.0, 87.2	Homicide offense, psychiatric referrals	NC *Detained in hospital: court referrals to hospital, referred to as “criminal remands”	Psychiatric record (D), questionnaire (S)	84.61% schizophrenia or psychosis; 2.56% personality disorder; 12.82% epilepsy	Medium
Menezes et al. 2007 [113]	Prevalence	Forensic ward	Zimbabwe, England, Wales	Y (referral patients in England and Wales)	Census	Yes, Yes	367, 36.0, 91.8% (Zimbabwe); 1966, 29.7, 83.6% (England/Wales)	Psychiatric referrals	U *Referrals to hospital	Psychiatric record, ICD-9 criteria (D), questionnaire (S)	78.7% of patients in Zimbabwe had a mental disorder diagnosis compared with 51.5% in England and Wales (p < 0.001). 6.3% had personality disorder diagnosis in Zimbabwe; 36.6% in England and Wales	Medium
Odejide 1981 [114]	Prevalence	Forensic ward	Nigeria	N	Census	No, No	53, 38.7, 83%	Psychiatric referrals	U *Referrals to hospital	PSP (D)	75.5% schizophrenia; 5.7% drug-induced psychosis; 18.9% epilepsy (PSP)	Medium
Offen et al. 1986 [115]	Prevalence	Forensic ward	South Africa	N	Census	No, No	162, 20–40, 0%	Psychiatric referrals	U *Referrals to hospital	Psychiatric record (D)	82% had psychiatric abnormality, including 34% of total sample with significant psychiatric findings, but these were not considered of a critical enough nature to warrant the label “mental illness.”	Medium
Ogunlesi et al. 1988 [116]	Prevalence	Forensic ward	Nigeria	N	Census	No, No	146, 34.5, 98%	Psychiatric referrals	NC *Pre-trial referrals to hospital. Not convicted at time of diagnosis, but later conviction data provided	Psychiatric record (D)	45% schizophrenia; 4% mania; 3.3% depression; 0.7% paranoid state; 19.5% total drug abuse/dependence; 16.8% cannabis abuse; 2.7% alcoholism; 6.7% epilepsy. 75% had a previous history of psychiatric disorder; 45% admitted a previous history of drug abuse. 48% judged “criminal lunatics” either not guilty by reason of insanity or guilty but insane. 30% discharged by courts; 1 sentenced to death; 1 sentenced to a prison term. 46.3% of offenders absconded from the institution	Medium
Prinsloo and Hesselink 2014 [117]	Prevalence	Forensic ward	South Africa	N	Purposive, NS	No, No	91, NS, 100%	Psychiatric referrals	NC *Pre-trial referrals to hospital	Psychiatric record (D)	83.5% at least one mental health disorder	Medium
Strydom et al. 2011 [54]	Prevalence	Forensic ward	South Africa	N	Census	Yes, No	120, 32.5, 95.8%	Psychiatric referrals	NC *Detained “state patients” accused but found unfit to stand trial or not responsible, referred to forensic ward	Psychiatric record (D)	Most had a history of abusing substances such as alcohol (74%), cannabis (66.7%), tobacco (29.6%) and glue (6.2%). 55.5% diagnosed with schizophrenia; 9.2% bipolar mood disorder; 5.9% psychosis due to general medical condition; 4.2% psychosis due to epilepsy; 3.4% psychosis due to substance abuse; 1.7% delirium; 10% other disorder	Medium
Touari et al. 1993 [118]	Prevalence	Forensic ward	Algeria	N	Census	No, No	2882, 30.1, 94.3%	Psychiatric referrals	NC *Pre-trial	Psychiatric record (D)	11.1% diagnosis of psychosis. 1.4% diagnosis of manic depression	Medium
Turkson and Asante 1997 [55]	Prevalence	Forensic ward	Ghana	N	Census	No, No	130, NS, 94.6%	Psychiatric referrals and state patients	NC *Detained in hospital: Pre-trial, convicted, or found unfit to stand trial. Participants were “predominantly patients who had been found guilty but insane or those found unfit to proceed with their trial” due to “insanity”	Psychiatric record (D) and clinical observation by author (S)	81.6% had a psychiatric diagnosis as indicated by clinical records. At the time of the study, 70.9% of total patients exhibited no florid psychotic symptoms, all patients with a diagnosis of harmful drug use were free from symptoms; 93.8% diagnosed with drug-induced psychosis were fully recovered	Medium
Verster and Van Rensburg 1999 [119]	Prevalence	Forensic ward	South Africa	N	Census	Yes, No	126, NS, 98.4%	Have homicide offense and psychiatric referrals	NC *Pre-trial referrals to hospital	Psychiatric record (D)	42.1% had a psychiatric diagnosis	Medium
Yusuf and Nuhu 2009 [120]	Prevalence	Forensic ward	Nigeria	N	Census	No, No	19. 28.9, 73.7%	Psychiatric referrals	NS	Psychiatric record (D)	Schizophrenia was the most common psychiatric disorder (68.4%), co-morbid substance use present in 57.9%	Medium
Zabow 1989 [121]	Prevalence	Forensic ward	South Africa	N	Census	No, No	202, NS, 90%	Homicide convicts	NC *Pre-trial referrals to hospital	Psychiatric record (D)	15.8% prevalence of “significant psychiatric findings.” Alcohol and drugs were contributory to the criminal behavior in 50% of cases. The number of murders committed increased by 25.2% in 1977–1984 compared to an increase of 115.8% in the number of psychiatric referrals during the same period. Following hospital assessment, 60.4% had no psychiatric diagnosis	Medium
Atilola et al. 2014 [7]	Prevalence	Youth Institution	Nigeria	Y (school-going adolescents, age matched but school-going youth slightly younger. Detained youth 18.7 ± 2.4 years old [Range 16–20 years] vs. school kids 18.2 ± 2.5 [Range 15–19 years])	Census	Yes, No	144, 18.7, 100% (participants in Borstal home); 144, 18.2, 100% (school-going youth)	NA	JI *Detained in borstal institution in juvenile justice system: classified 52.1% juvenile offenders; 47.9% youth beyond parental control (no offense)	K-SADS-PL (D)	90% of the justice-involved youth in borstal home reported exposure to at least one lifetime traumatic event, compared with 60% of the comparison group (p = 0.001). Justice-involved youth also had a higher mean number of incident lifetime traumatic events (p < 0.001), and higher prevalence rate of current and lifetime PTSD than the comparison group (p < 0.05). Justice-involved more likely to be victims of violent crime (p < 0.001), have experienced physical abuse (p < 0.001), and be perpetrators of a violent crime (p = 0.002) (K-SADS-PL)	Low
Atilola 2012 “Different points…” [122]	Prevalence	Youth Institution	Nigeria	Y (within-institution comparison of youth on criminal code vs. youth in care of state/neglected youth)	Census	Yes, Yes	158, 17.5, 96. % (criminal code group); 53, 12.5, 74% (in care of state)	NA	JI *75% criminal code or beyond parental control, 25% due to maltreatment/neglect	K-SADS (D)	Conduct/behavior disorders had 63% prevalence among “criminal code” youth vs. 39%, among neglect group (p < 0.001). Prevalence of multiple traumatic events 27% among criminal code youth; 26%, neglect group (p = 0.43). PTSD prevalence 13% among criminal code youth; 22% among neglect group (p = 0.12). Substance use prevalence was 61% among those on criminal code compared to 11% youth detained due to neglect/maltreatment (p = 0.003) (all K-SADS)	Medium
Atilola 2012 “Prevalence and correlates…” [6]	Prevalence	Youth Institution	Nigeria	Y (school-going adolescents, age and gender matched, randomly selecter)	Census	Yes, Yes	60 (in remand home), 60 (school-going), 12.5* (pooled), 66.6%* (pooled) *Only pooled statistics given	NA	NC *77% in home due to maltreatment/neglect, 10% classified as “offenders,” 13% beyond parental control	K-SADS-PL (D)	63% remanded participants had at least one lifetime psychiatric disorder compared to 23% control (p < .001); 22% had at least one current psychiatric disorder compared to 3% control (p < .004) (K-SADS-PL)	Medium
Atilola et al. 2016 [50]	Prevalence	Youth Institution	Nigeria	Y (within-institution comparison of “criminal code” vs. other groups)	Random, NS	Yes, Yes	178, 15.19, 61.8% (total participants, pooled)	NA	NC *19.1% classified “young offenders,” 73.6% care and protection of state, 7.3% beyond parental control	K-SADS (D)	Lifetime prevalence rate of abuse of/dependence on any substance was 22.5%. 12.3% alcohol abuse/dependence; 17.9% other substance abuse/dependence. Higher proportion of participants who were remanded under the ‘young offender’ category met criteria for lifetime substance use disorder compared with those under the care and protection and beyond-parental-control category (p = 0.004). Length of staying on the streets or by self was associated with problematic use (abuse or dependence) (p = 0.007) (K-SADS)	Low
*Atilola et al. 2017 “Correlations…” [123]	Prevalence	Youth Institution	Nigeria	N	Random, NS	Yes, Yes	165, 14.3, 75%	NA	NS *Remanded youth: criminal code, neglected/in care of state, or beyond parental control	SDQ (S), PedsQo (S)	18% abnormal SDQ score suggesting presence of psychiatric disorder; 27% had ‘highly probable’ psychopathology (SDQ). Negative correlation (p < 0.001) between total SDQ scores and overall self-reported quality of life (PedsQo)	Low
*Atilola et al. 2017 “Status…” [124]	Prevalence	Youth Institution	Nigeria	N	Random, NS	Yes, Yes	165, 14.3, 75.2%	NA	NS *Remanded youth: criminal code, neglected/in care of state, or beyond parental control	SDQ (S), CRAFFT (S), questionnaire (S), Audit Protocol (S)	18.2% general psychiatric morbidity by SDQ ≥ 17; 44.6% prevalence SDQ ≥ 15; 15.8% alcohol/substance use disorder (CRAFFT > 2). 34.3% of the operational staff at the institutions had educational backgrounds relevant to psychosocial services for children/adolescents. Less than a quarter (22.4%) ever received any training in child mental health services (questionnaire and Audit protocol)	Low
*Adegunloye et al. 2010 [125]	Prevalence	Youth Institution	Nigeria	N	Census	No, No	53, 17.3, 100%	NA	JI * Detained in borstal institution in juvenile justice system	GHQ-12 (S), MINI-KID (D)	67.9% current psychiatric disorder (MINI-KID). GHQ scores not reported	Low
*Ajiboye et al. 2009 (same sample as Adegunloye) [126]	Prevalence	Youth Institution	Nigeria	N	Census	No, Yes	53, 17.3, 100%	NA	JI * Detained in borstal institution in juvenile justice system	GHQ-12 (S), MINI-KID (D)	67.9% current psychiatric disorder (MINI-KID). GHQ scores not reported	Low
*Issa et al. 2009 (same sample as Adegunloye) [127]	Prevalence	Youth Institution	Nigeria	N	Census	Yes, Yes	53, 17.3, 100%	NA	JI * Detained in borstal institution in juvenile justice system: classified “juvenile offenders” or those “in need of correction”	GHQ-12 (S)	49.1% GHQ-positive (> 3 on GHQ-12), indicating possible psychiatric morbidity	Medium
*Yusuf et al. 2011 (same sample as Adegunloye) [128]	Prevalence	Youth Institution	Nigeria	N	Census	No, Yes	53, 17.3, 100%	NA	JI * I Detained in borstal institution in juvenile justice system	GHQ-12 (S), MINI-KID (D)	50.9% had MINI-KID lifetime psychiatric diagnoses. Majority (62.3%) had psychiatric problems in the past 12 months. When all lifetime and current psychiatric diagnoses were collapsed, 98.1% had ‘any psychiatric disorder. 49.1% GHQ-12 > 3, indicating possible psychiatric morbidity	Low
Bella et al. 2010 [51]	Prevalence	Youth Institution	Nigeria	N	NS	No, Yes	59, 11.7, 60%	NA	NC *90% under care and protection of state, 7% beyond parental control, 3% criminal code/“youth offenders”	K-SADS (D)	100% had significant psychosocial needs presenting as difficulty with their primary support, social environment, or education systems. 97% demonstrated some form of psychopathy	Medium
*Olashore et al. 2016 [129]	Prevalence	Youth Institution	Nigeria	N	Census	Yes, Yes	148, 17.1, 100%	NA	JI * Detained in borstal institution under criminal code or beyond parental control; 40.8% detained for “non-delinquent reason”	MINI-KID (D)	56.5% met the criteria for conduct disorder (MINI-KID). Number of siblings (p = 0.010) and previous history of detention (p = 0.043) were independent predictors of CD	Low
*Olashore et al. 2017 [130]	Prevalence	Youth Institution	Nigeria	N	Census	Yes, Yes	148, 17.1, 100%	NA	JI * Detained in borstal institution under criminal code or beyond parental control; 40.8% detained for “non-delinquent reason”	MINI-KID (D)	56.5% met the criteria for conduct disorder (MINI-KID). Substance use, depression, or oppositional defiant disorder not significantly associated with “offender” status. CD is associated (p < .001) with “offender” status	Low

**Table 3:** All other study designs

Reference *If same sample as another study in list	Study design	Study Setting	Country setting	Comparison [If yes (Y), describe; no (N)]	Strategy, Whether sample size calculation was reported for non-census strategies	Ethics reporting (documented ethics committee approval; described informed consent procedure)	Participants characteristics (sample size, mean age, percent male) *Indicates gender as inclusion criteria	Inclusion criteria (excluding age criteria)	Trial status category (*1) (C = over 50% convicted; NC = over 50% not convicted; NC/A = over 50% “awaiting trial”; JI = over 50% youth justice-involved; U = unclear; NA = not applicable; NS = not stated)	Assessment instruments (diagnostic or screening tool)	Primary outcomes (p-value listed if provided in study)	Methods risk of bias score
Eseadi et al. 2017 [42]	Pre-post	Prison	Nigeria	Y (15 treatment, 15 control group not receiving intervention)	Census	Yes, Yes	15, NS, 100% (treatment); 15, NS, 100% (control)	BDI score ≥ 29	NS *But 84% awaiting trial in the prison population from which the sample was selected	BDI (S)	Significant treatment by time interaction effect for cognitive behavioral coaching program on depression as measured by BDI (p = 0.000). Significant decrease from pre to post-test BDI score (p = 0.000) for the CBC group compared to control	Low
Onyechi et al. 2017 [43]	Pre-post	Prison	Nigeria	Y (10 treatment, 10 control group not receiving intervention)	Census	Yes, Yes	10, NS, 100% (treatment); 10, NS, 100% (control)	High scorers on CDS-12	NS	CDS-12 (S)	After the cognitive behavioral intervention, prisoners in the treatment group has significantly lower post-intervention CDS-12 scores than the control group’s post-intervention scores (p = 0.00)	Medium
Martyns-Yellowe 1993 [44]	RCT	Prison	Nigeria	Y (18 participants each in treatment groups receiving Flupenthixol or Clopenthixol injections)	Census	No, No	18, NS, 100%* (Flupenthixol treatment); 18, NS, 100%* (Clopenthixol treatment)	Males; schizophrenia diagnosis; vagrant people removed from public places by law enforcement	U *Detained in prison asylum after “removed from streets”	BPRS (Brief Psychiatric Rating Scale) (S)	57.1% drop in BPRS symptoms in the Flupenthixol group (p < 0.001) and 43.4% drop in the Clopenthixol group (p < 0.01). Flupenthixol group had better symptom reducation respsone than the Clopenthixol group (p < 0.01)	Medium
Balogun and Olawoye 2013 [40]	Cross-sectional	Prison	Nigeria	Y (within institution comparison of high/low self-esteem and high/low emotional intelligence)	NS	No, No	233, 31.3, 86.27% (total participants)	NA	NS	SDS Self-Rating Depression Scale (S), TMMS Trait Meta-Mood Scale (S), Rosenberg self-esteem scale (S)	Both emotional intelligence (p < 0.05) and self-esteem (p < 0.05) had a significant influence on depression	Low
Idemudia 1998 [131]	Cross-sectional	Prison	Nigeria	N	Random, NS	No, No	150, 27.8, 61.3%	NA	NS	API (S), MSQ/CCEI (S)	Long-term detained persons had higher mean scores of psychopathy symptoms (API), (p < 0.001), and neurotic symptoms (MSQ/CCEI), (p < 0.001), than those serving medium and short terms	Medium
Idemudia 2007 [132]	Cross-sectional	Prison	Nigeria	Y (college students, matched for gender, youth characteristic, and age*) *However, we note that statistics show that college students have noticeably older mean age	Random, NS	No, No	100, 17.2, 83% (detained participants); 100, 25.2, 81% (college students)	Homeless on street before prison	NS	PDS (S), MAACL-H (S)	Higher scores on the Psychopathic Deviate Scale (p < .05) and the Multiple Affect Adjective Checklist hostility subscale (p < .0001) among the imprisoned homeless group than the non-prison and never homeless group	Medium
Ineme and Osinowo 2016 [133]	Cross-sectional	Prison	Nigeria	N	Random, NS	Yes, Yes	212, 34.4, 86.3%	NA	NS	HADS (S), IS-HUS (S), questionnaire (S)	Participants who used psychoactive substances (questionnaire) before detention reported higher self-harm urges (IS-HUS) than those who did not use (p < .01). Participants with higher depressive symptoms (HADS) reported higher self-harm urges than those with low depressive symptoms (p < .01}. Significant interaction of prior substance use and depression (< .01)	Low
Stephens et al. 2006 [38]	Cross-sectional	Prison	South Africa	N	Census	Yes, Yes	357, NS, 100%*	Males; pre-release; scheduled to be released from prison within three months after receiving intervention in parent study	U *all participants have pre-release status	Questionnaire (S)	Participants who used psychoactive substances (questionnaire) before detention reported higher self-harm urges (IS-HUS) than those who did not use (p < .01). Participants with higher depressive symptoms (HADS) reported higher self-harm urges than those with low depressive symptoms (p < .01}. Significant interaction of prior substance use and depression (< .01)	Medium
Weierstall et al. 2011 [37]	Cross-sectional	Prison	Rwanda	N	Random, NS	Yes, Yes	269, 33, 66%	Perpetrators of the Rwandan genocide	C *82% convicted, 18% awaiting trial	PTSD Symptom Scale-Interview (PSS-I) (D), PDS Event Scale (S), Appetitive Aggression Scale (AAS) (S)	Dose–response effect via path analysis between the exposure to traumatic events and the PTSD symptom severity (p < .001). Participants who had reported that they committed more types of crimes demonstrated a higher AAS score (p < .01), and higher AAS scores predicted lower PTSD symptom severity scores (p < .05).	Low
Odejide 1979 [134]	Cross-sectional	Forensic ward	Nigeria	N	Census	No, No	2158, NS, 95.9%	Psychiatric referrals	U *Referrals to hospital	Court records (NA)	32.4% of 81 individuals with murder charges were referred for psychiatric opinion. No individuals with charges in categories of crime, including three individuals with charges of attempted suicide, was sent for psychiatric examination. Absence of mental illness in 66.6% of subjects referred for psychiatric opinion	Low
Sukeri et al. 2016 [135]	Cross-sectional	Forensic ward	South Africa	N	Census	No, No	NA	NA	NA	Questionnaire (S)	No nurses with advanced training in forensic psychiatry. Lack of sufficient human resources. The nurse/patient ratio was 1:4. For 403 patients, 1.6 psychiatrists (1 full time),1 social worker, 1 occupational therapist, 0 occupational therapist assistants. There are 22 psychologists in all correctional centers in South Africa. None of the correctional centers have an onsite psychiatric unit	Low
Ononye and Morakinyo 1994 [39]	Cross-sectional	Youth Institution	Nigeria	Y (50 school going children, matched for sex, age, ethnicity and educational level)	Census	No, No	50, 14.1, 86% (youth in remand home); 50, 14.1, 86% (school-going youth)	NA	NS *Remanded youth	Carlson Psychological Survey (CPS) (S)	Thought disturbance significantly higher in youth in remand home compared to school-going youth. Antisocial tendency and self-depreciation higher among youth in remand home but not significantly. Substance abuse not significantly different between groups. (all indicated by CPS)	Medium
Large and Nielssen 2009 [41]	Cross-sectional	Health system	International	Y (LMIC and HIC countries)	Census	No, No	NA	NA	NS	Published records in the literature (NA)	Correlation between per capita psychiatric hospital beds and prisoner numbers in the 158 countries (p < 0.01) and the subgroup of 120 LAMI countries (p < 0.01). No significant correlation within the 38 HI countries	Low
Gaum et al. 2006 [45]	Qualitative	Prison	South Africa	N	Convenience, N	No, Yes	10, 37.6, 50% (interviews); 18, NS, 100% (in focus groups)	Recidivists; psychological services clients	C	Interviews and focus groups	Interviews reveal a shortage of medical personnel in the prison psychiatry/psychology service. Also suggested from interviews: overpopulation in prisons may be due to rapid and dramatic political and economic changes in South Africa, coupled with the belief that crime pays and that being in prison is preferable to being jobless and homeless outside	Low
Pretorius and Bester 2009 [47]	Qualitative	Prison	South Africa	N	Purposive, N	Yes, Yes	3, 35–42, 0%	Women convicted of homicide of their intimate partner	C	Interview	All three participants’ interviews were indicative of PTSD and substance misuse	Low
Topp et al. 2016 [46]	Qualitative	Prison	Zambia	N	Purposive and Random, N	Yes, Yes	79, 35.6, 100%* (detained); 32, NS, 50% (prison staff)	Detained men	C *70–100% convicted depending on facility	Interviews and focus groups	A majority of participants in prison, as well as facility-based officers reported anxiety linked to over-crowding, sanitation, infectious disease transmission, nutrition and coercion. Interviewees associated overcrowding with negative effects on both participants in prison and officers’ physical and mental health. Limited access to healthcare	Low
Kaliski et al. 1997 [48]	Qualitative	Forensic ward	South Africa	N	Census	No, Yes	88, 30.4, 100%	Defendants undergoing psychiatric referral	NC *Pre-trial defendants for psychiatric observation	Psychiatric record (D)	30.7% ultimately declared mentally ill. Only 25% knew that they were to be psychiatrically examined during the 30-day period. 44.3% did not know what was to happen to them after the completion of the observation period	Low
Dube-Mawerewere 2015 [49]	Structured health system review	Health system	Zimbabwe	N	Purposive, N	No, No	32, NA, NA	Forensic psychiatry system stakeholders	NS	Interview	Special psychiatric institutions housed within prisons, resulting in prison-like living conditions. Lack of staff in special institutions and forensic psychiatry settings with psychiatric training. Revolving door between civil psychiatric institutions in the prison, forensic hospital, and prison	Not assessed due to study design
Kidia, et al. 2017 [22]	Structured health system review	Health system	Zimbabwe	N	Purposive, N	No, No	30, NA, NA	Mental health system stakeholders, excluding patients	NA	Interviews, Emerald national-level needs assessment methods	Forensic facilities were substantially under-resourced, especially shortages of psychotropic medicines and human resources. Patients lived in overcrowded holding cells with unhygienic living conditions, with high prevalence of sexual assault and HIV transmission, minimal access to psychotropic medications and psychiatric care, and little food	Not assessed due to study design
Liddicoat et al. 1972 [136]	Tool validation	Prison	South Africa	Y (99 participants with psychopathy diagnosis and 99 without psychopathy diagnosis matched for age and IQ)	Purposive, NS	No, No	198, NS, 100% (total participants, pooled)	Participants with and without psychopathy diagnosis	C	Questionnaire (S)	64/150 items on the questionnaire discriminated significantly between participants with and without psychopathy diagnosis	Not assessed due to study design
Prinsloo and Ladikos 2007 [137]	Tool validation	Prison	South Africa	Y (231 those with offense designated “high-risk” compared to 38 segregated due to history of maladjustment, disciplinary problems and other institutional infractions)	Purposive, NS	No, Yes	269, 31.8, 100%* (total participants, pooled)	Men with offense; those designated “high-risk”	NS	SAQ (S)	The overall alpha score of the SAQ, inclusive of all the interactive subscales, is (.904)	Not assessed due to study design
Prinsloo 2013 [138]	Tool validation	Prison	South Africa	N	NS	No, Yes	236, 34, 100%	NA	C	Psychiatric record (D), SAQ (S)	Logistic regression model of the behavioral characteristics assessed with the Self-Appraisal Questionnaire (SAQ) shows that modeling the behavioral characteristics accounts for 61% of the variation in the dependent variable mental illness. Subscales of anger, criminal tendencies and anti-social personality have significantly higher (p < 0.05) mean scores for mentally ill respondents	Not assessed due to study design
Bunnting et al. 1996 [139]	Tool validation	Forensic ward	South Africa	Y (50 patients designated “malingering” and 50 state patients with mental disorder or sick (State President’s Detainees)	Purposive, N	No, No	100, NS, NS (total participants, pooled)	Psychiatric referrals and state patients	NS *Pre-trial, convicted, and referrals	Questionnaire (S)	17/20 items on the questionnaire statistically significant based on the study sample	Not assessed due to study design

### Data analysis

Because of the heterogeneity of the study designs and outcomes, we conducted narrative analysis, as an overall meta-analysis was not possible. To facilitate comparison between like studies, we structured our presentation of results by study design.

### Meta-analysis

The large number of prevalence studies enabled us to conduct a meta-analysis of the prevalence of mental disorders. Notably, this analysis was added post hoc, as we did not anticipate sufficient study design homogeneity for meta-analysis at the outset of the study. We generated pooled prevalence estimates with 95% confidence intervals [32] for key disease categories: mental ill health (including measures of psychological distress or an unspecified mental disorder, often assessed using a screening tool, such as the General Health Questionnaire (GHQ) [33], but excluding results of disorder-specific instruments), mood disorders, psychotic disorders, and substance use. Heterogeneity between studies within each category was assessed using Chi squared and I^2^ (> 50% is considered heterogeneous [34]). A random effects model was used to estimate the pooled prevalence, as all groups demonstrated significant heterogeneity. The random effects model weights included studies to account for both sample size and between-study variance with the between-study variance term dominating the weighting when studies are heterogeneous [35], as it assumes that studies come from different distributions. In order to identify sources of heterogeneity, we conducted subgroup analysis, grouping according to youth and adults; then, within studies of adults, location (prison or forensic ward) and data collection method (instrument or clinical record). In the subgroup analysis, we determined the pooled prevalence for each subgroup and its heterogeneity. Statistical analysis was done using STATA/SE 15.0 [36]. Additional details of the data extraction and statistical analysis are shown in Additional file 1: Appendices S4, S6 and S7.

## Results

### Search results

After removing duplicates, our search yielded 1240 results in the initial database search, of which 73 met inclusion criteria. We excluded three studies based on the methods screen results and added eight from a backward search. We were not able to access the texts or abstracts of three studies from the backward search title results because they were not available for loan from multiple university library systems. A third researcher (HJ) reviewed 39 full texts about which the other two reviewers could not reach agreement and chose to include 12 of them (31%). We added two articles identified through expert consultation. This yielded 80 papers for data extraction (Fig. 1), including 17 papers that were written on the same sample and as part of the same study as another paper in our dataset, but with different methods details or outcomes reported. In this review, the terms “independent studies” or “samples” refer to the number of independent studies, and the term “papers” refers to all papers in the dataset. There were 70 independent studies in our data. All papers are described in Tables 2 and 3.

### Study characteristics

Prevalence was the dominant study type (67%), with small numbers of other study types including ten non-prevalence cross-sectional studies, four qualitative studies, four tool validations, two structured health systems reviews, two pre-post studies, and one randomized control trial. The majority of studies took place in either Nigeria (30; 43%) or South Africa (21; 30%). Only five of 23 non-prevalence studies took place outside of Nigeria or South Africa: two health systems studies in Zimbabwe, one qualitative study in Zambia, one cross-sectional study in Rwanda, and one international comparative study. Sixty-nine percent of studies collected primary data, or data resulting from diagnostic or screening tools, and 37% collected secondary data, defined as data extracted from available records. Four studies collected both primary and secondary data. Thirty-two studies (46%) examined prison settings, of which all but one study collected primary data. Twenty-six studies (37%) examined forensic hospital settings (included only if they were settings in which a justice-involved population was detained). Among these, 88% were based on secondary data, and the majority took place in either South Africa (54%), Nigeria (15%), or Zimbabwe (12%). Nine studies (13%) examined youth institutions, of which all were conducted in Nigeria. Three studies (4%), two conducted in Zimbabwe and one international, examined the mental health system.

### Risk of bias

Most of the papers assessed fell into the low (36; 49%) or medium (33; 45%) methods risk of bias categories. A number of medium risk papers were given this judgement based on lack of detail in methods reporting, meaning that it was unclear if the studies had high risk of bias or if methods were not reported well. About half of the medium risk papers reported diagnoses from secondary psychiatric records (17), for which it was often unclear how the diagnoses were made, who made them, or if they were based on standard criteria, such as the ICD. Of the 57 psychiatric prevalence papers, 44% (25) fell into low risk, 47% (27) fell into medium, and 9% (5) fell into high risk of bias categories. All “high risk” studies were prevalence papers. See Additional file 1: Appendix S5 for results of the methodologic review of prevalence studies, which includes evaluation of sampling technique using the Prevalence Critical Appraisal Checklist.

### Data collection method

Most studies used a census sampling strategy (42; 60%), followed by random (18; 26%), purposive (8; 11%), not stated (4; 5.7%), mixed sampling (3; 4.3%), and convenience sampling (1, 1.4%). Of all psychiatric prevalence papers, 60% used validated instruments for primary data collection, while the rest extracted data from secondary psychiatric records.

### Participants

In 69% of samples, more than 85% of participants were male. In 10 samples, “male” was an inclusion criterion. The mean age of participants among samples listing mean age was 28.8, and there was a broad distribution of sample size, from 18% of participant samples with 50 people or less (including all three interventions), to 13% with over 400 participants. In 36% of samples, the majority of participants had not been convicted of any crime (the majority of participants were either awaiting trial or detained without trial). In another 41% of samples, trial status was not stated or unclear, or participants were justice-involved youth (in which trial status was ambiguous). In only 20% of samples, the majority of participants had been convicted.

### Ethics characteristics

Of all papers, 35% neither documented ethics committee approval nor described an informed consent procedure, and 41% described both. Ten percent reported ethics committee approval but not informed consent, and 14% reported informed consent but not ethics committee approval.

### Outcomes

To facilitate comparison between like outcomes, we have presented study outcomes by study design: prevalence, non-prevalence cross-sectional, intervention testing, qualitative, structured health system review, and tool validation. Of the 57 prevalence papers reporting mental health diagnoses or screening results, 44 (77%) reported diagnoses of psychiatric conditions, of which 14 papers also used instruments designed to screen for psychiatric morbidity but not diagnose. Thirteen papers (23%) reported outcomes obtained with screening tools alone, not diagnostic instruments. Reported factors associated with a psychiatric outcome and secondary outcomes are displayed in Additional file 1: Appendices S10 and S11.

### Prevalence studies

**Fig. 2:**
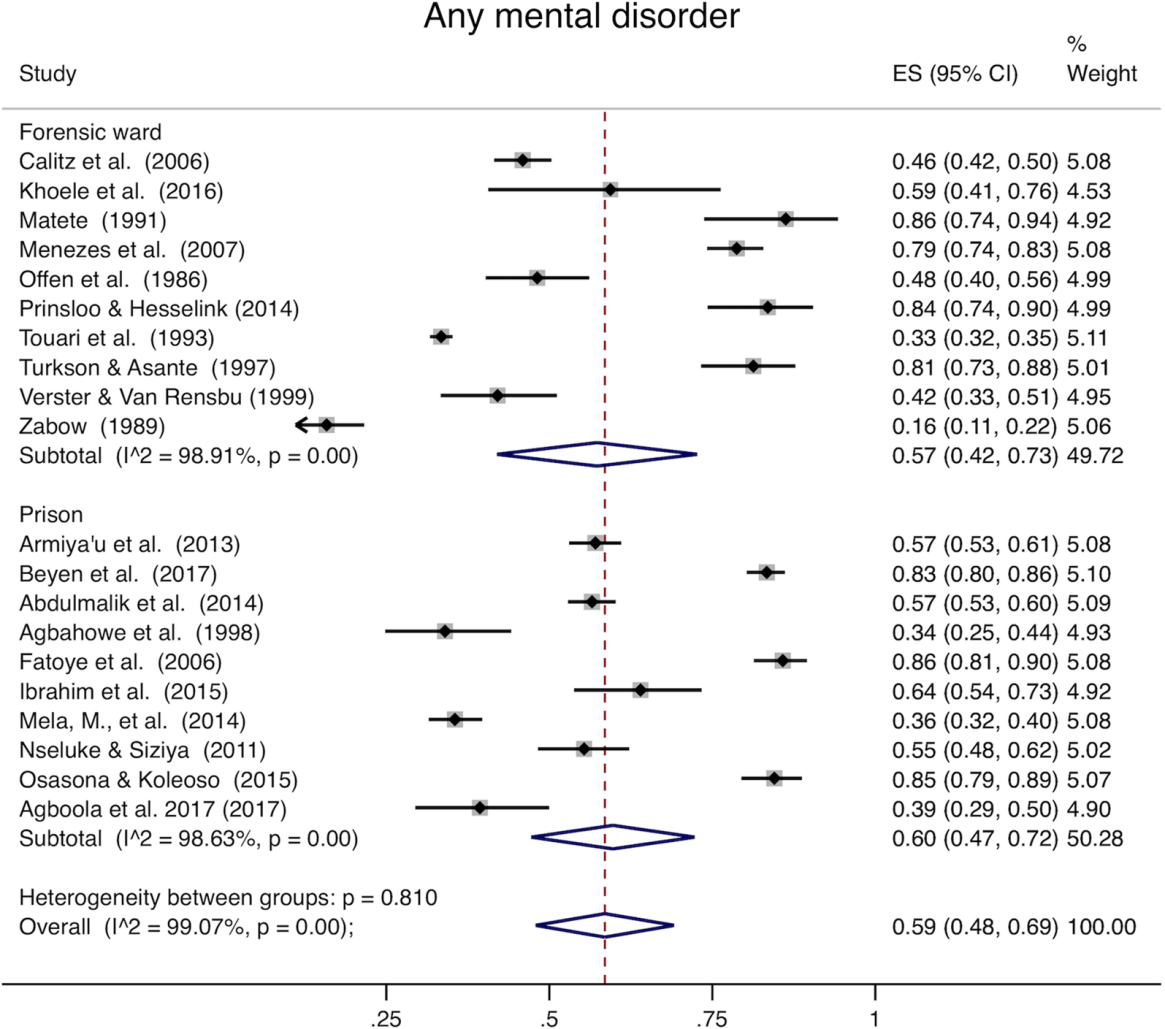
Mental ill health. The x-axis in each plot displays prevalence (0–1). The far right column displays the prevalence within each study, pooled prevalence for each subgroup, and overall pooled prevalence with their respective weights. Armiya’u et al. [86] and Armiya’u et al. [87] describe the same study and sample and report the same prevalence. Only one point prevalence from this sample was included from these articles in Figs. 2, 3, 4, and 5

**Fig. 3:**
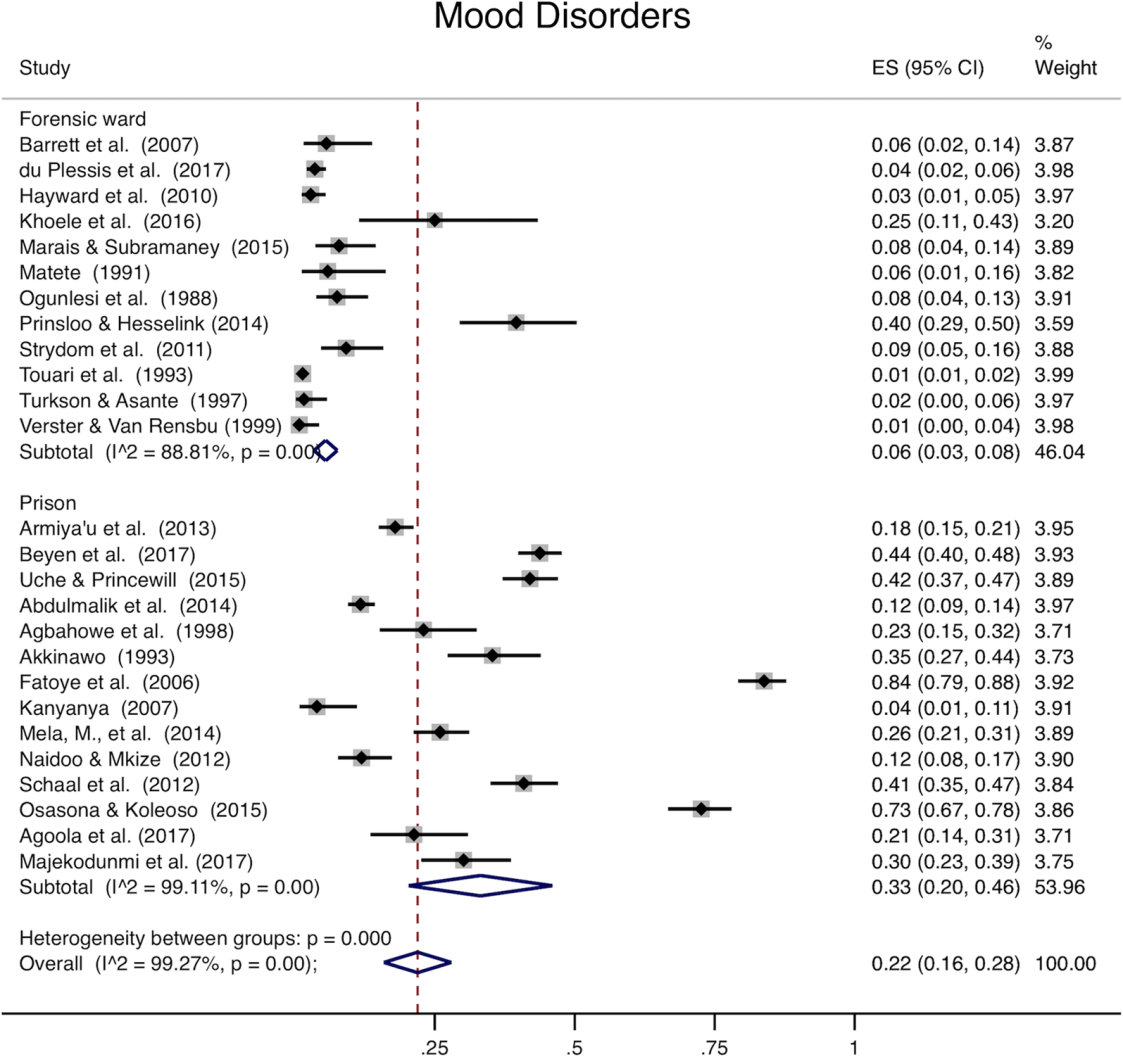
Mood disorders. The x-axis in each plot displays prevalence (0–1). The far right column displays the prevalence within each study, pooled prevalence for each subgroup, and overall pooled prevalence with their respective weights. Uche and Princewell “Prevalence…” (2015) [103] and Uche and Princewell “Clinical factors…” (2015) [102] describe the same study and sample and report the same prevalence. Only one point prevalence from this sample was included from these articles

**Fig. 4:**
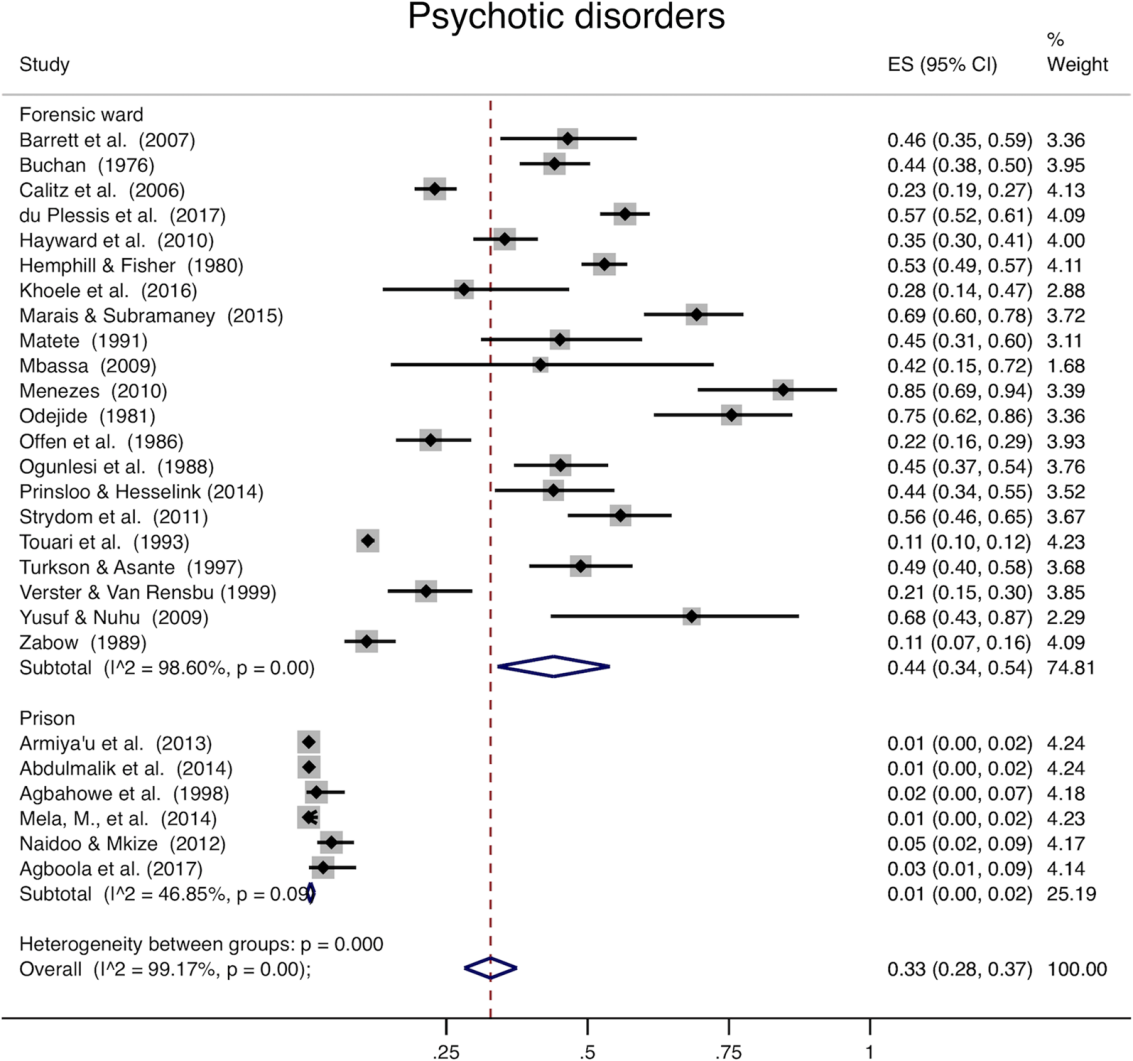
Psychotic disorders. The x-axis in each plot displays prevalence (0–1). The far right column displays the prevalence within each study, pooled prevalence for each subgroup, and overall pooled prevalence with their respective weights

**Fig. 5:**
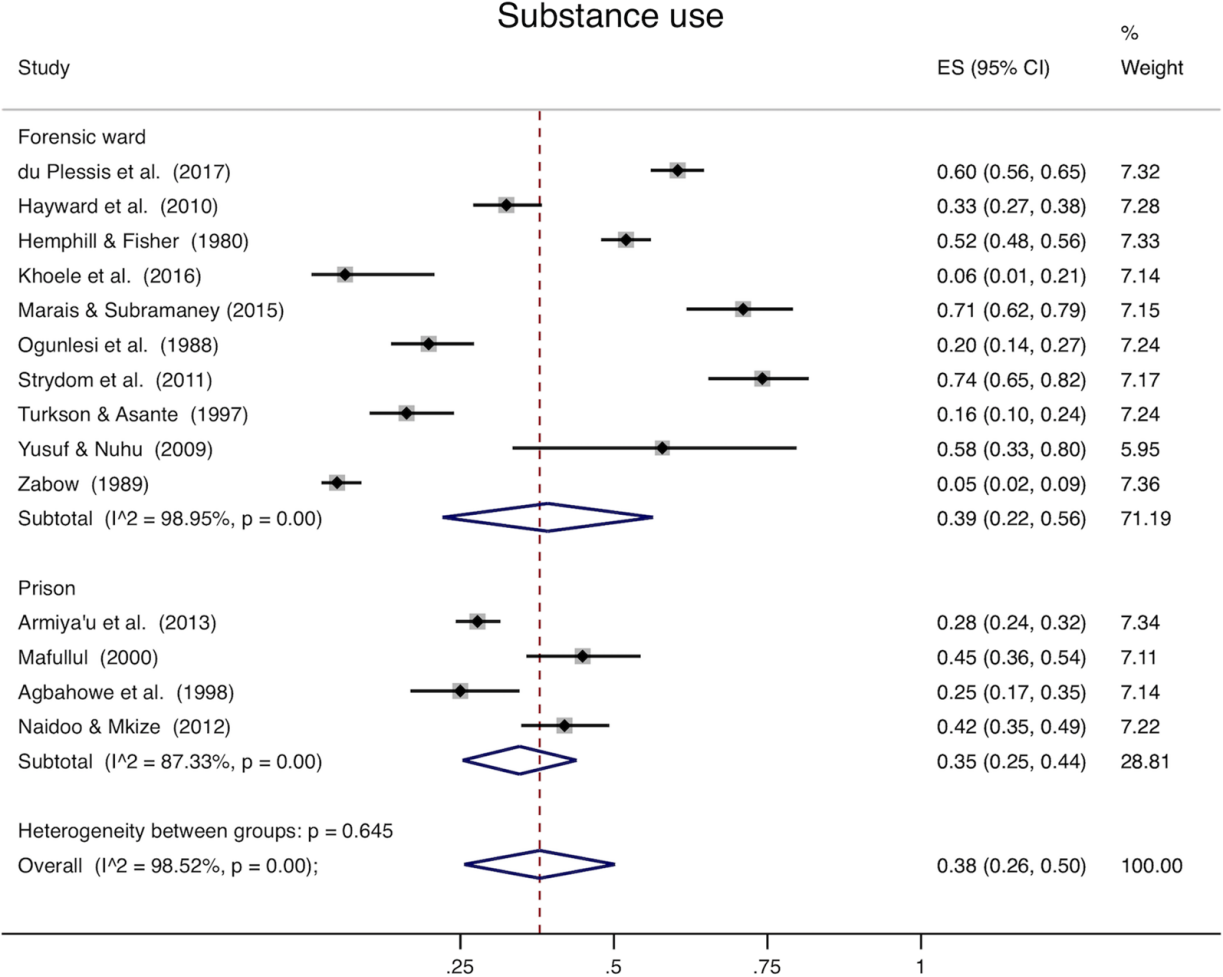
Substance use. The x-axis in each plot displays prevalence (0–1). The far right column displays the prevalence within each study, pooled prevalence for each subgroup, and overall pooled prevalence with their respective weights

Analysis of all studies combined (Table 4) was heterogeneous (I^2^ > 98% for all disease categories). When youth and adults were examined separately, pooled prevalence among adults was 59% for mental ill health (95% CI 48–69%, Fig. 2), 22% for mood disorders (95% CI 16–28%, Fig. 3), 33% for psychotic disorders (95% CI 28–37%, Fig. 4), and 38% for substance use (95% CI 26–50%, Fig. 5). Among youth (Table 5), prevalence of mental ill health was 61% (95% CI 17–100%), mood disorder was 24% (95% CI 14–25%), and substance use was 22% (95% CI 8–36%). Heterogeneity analysis revealed statistically significant heterogeneity for all disease categories and subgroup by institution (Figs. 2, 3, 4 and 5) (I^2^ > 50%). The one exception was psychotic disorders in prisons, which because of their very low prevalence, were less heterogeneous (I^2^ = 46.85%). The prevalence of psychotic disorders among inpatients in forensic wards was 44% (95% CI 34–54%), while in prisons the prevalence was 1% (95% CI 0–2%). Subgroup analysis by data collection method (clinical record or diagnostic/screening instrument) was not presented separately; all of the studies conducted in prisons use instruments to collect data, and all but one of those conducted in forensic institutions use clinical records. Thus, there is so much confounding that we cannot meaningfully separate the effects of data collection method from institution type. Robustness analysis (Additional file 1: Appendix S6) showed that the point prevalence estimates were similar regardless of how the subgrouping was done. Additionally, to examine if there was heterogeneity based on sampling technique, we conducted a sensitivity analysis including studies using census sampling only (the most homogenous sampling technique; randomization can vary substantially based on the method used to randomize, and the randomization method was not stated in most included studies) (Additional file 1: Appendix S7). Prevalence estimates of this subgrouping had as much heterogeneity as estimates from pooling different sampling methods (census, random, not stated) and were similar to overall estimates.

**Table 4:** Overall prevalence of mental disorders

	Mental ill health	Mood disorder	Substance use	Psychotic disorders
Pooled prevalence (95% CI)	0.59 (0.49–0.69)	0.22 (0.16–0.28)	0.34 (0.24–0.44)	0.32 (0.27–0.36)
Heterogeneity chi^2^	2513.79 (p<0.001)	3447.07 (p<0.001)	1025.05 (p<0.001)	3133.52 (p<0.001)
I^2^	99.09%	99.19%	98.24%	99.14%
Number of studies	24	29	19	28

**Table 5:** Prevalence of mental disorders among youth

	Mental ill health	Mood disorder	Substance use
Pooled prevalence (95% CI)	0.61 (0.17–1.00*)	0.24 (0.14–0.35)	0.22 (0.08–0.36)
Heterogeneity chi^2^	422.59 (p<0.001)	4.93 (p=0.08)	95.75 (p<0.001)
I^2^	99.29%	59.46%	95.82%
Number of studies	4	3	5

Psychotic disorders were not included in this table, as only one youth study measured this outcome (Atilola 2012 “Prevalence and correlates…” [6])

*For clarity, this confidence interval is truncated at 1.00 as its upper bound

### Cross-sectional studies

Nine cross-sectional studies did not collect psychiatric prevalence data, but investigated variables associated with mental health conditions, or justice or health system qualities. These studies examined associations between mental health-related variables (for instance, PTSD symptom severity [37]; substance misuse [38, 39]) and other factors (crimes committed [37]; prevalence of sexually transmitted infections [38]; emotional intelligence [40]). Alternatively, large and Nielssen found a positive correlation between per capita prison populations and per capita psychiatric hospital beds among LMICs and a combined pool of 158 countries, but no significant correlation among high-income countries [41].

### Interventions

The intervention studies included two pre-post group-focused cognitive behavioral interventions in prisons, one for cigarette smoking dependence and one for depression, both yielding significant improvements in the treatment group compared to the control group (p < 0.001) [42, 43]. The single randomized control trial demonstrated significant decreases in schizophrenia symptoms following injections of two different neuroleptics in separate treatment arms (each neuroleptic treatment group resulted in a p < 0.01 decrease in the combined schizophrenia symptom score compared to the pre-injection score, and the Flupenthixol group showed a larger decrease than the Clopnethixol group (p < 0.01)) [44]. Additional details of the intervention studies are listed in Additional file 1: Appendix S8.

### Qualitative studies

The outcomes of the four qualitative studies included a shortage of medical personnel in prison mental health services in South Africa [45], poor prison conditions linked to mental health problems in a Zambian prison [46], psychiatric findings among women in prison for homicide [47], and gaps in awareness of the legal process and other legal characteristics of participants referred for psychiatric observation [48].

### Structured health systems reviews

The two structured health systems reviews, both in Zimbabwe, each interviewed around 30 participants. One used an exploratory qualitative design, including interviews with people detained in the justice system, and proposed a model for transforming the medico-judicial system that involves multiple stages of mental health screening and diversion from the justice system [49]. The other used a structured needs assessment and interviews with policy makers, administrators, providers, and researchers to examine the national mental health system and found that many stakeholders called attention to the forensic mental health system although the researchers did not specifically ask about forensic mental health [22].

### Descriptions of conditions of detention, policy, law, and health systems

We collected descriptions of mental health policy, laws, and systems if systematically collected and reported in any study type. Thirteen papers described conditions of detention in institutions, laws, or policy, including eight prevalence studies, three qualitative studies, and two structured health systems reviews. While it was not always clear whether the methodology used to report such outcomes was rigorous, we collected this data because of the scarcity of existing literature on this topic and report common findings thematically in Additional file 1: Appendix S9. The most common findings were insufficient human resources for health; lack of psychosocial services; lack of timely psychiatric assessment; and limited rehabilitation, recreational, vocational, or community re-integration services. Other common themes included insufficient physical resources, food, or psychiatric medicines; delays in trials, case-processing, or release; and lack of communication between medical and justice systems.

## Discussion

To our knowledge, this is the first systematic review to investigate the mental health of PDJS in Africa exclusively. Results reflect that existing studies on this topic are predominantly prevalence studies that show a high pooled prevalence of mental illness, consistent with previous findings on mental illness in detained populations globally. Notably, many people detained within the justice system in non-prison locations, such as youth institutions or forensic hospitals, were detained with no charge [6, 7, 50–55]. The neglect of these populations in the literature is especially alarming in the context of pressures to deinstitutionalize mental healthcare in LMICs [15, 56–59].

A number of key populations were missing from our results. There were very few women included in the included studies, reflective of the small proportion of women found in prisons worldwide, particularly Africa, where only 3% of the total prison population are female, much lower than elsewhere [60]. Reasons for this may be that worldwide, likely due to distinct social roles, women commit fewer crimes [61] and that women are less likely to be convicted of crimes and sent to prison by courts [62]. The studies were also concentrated in a small number of African countries (73% of studies were in South Africa or Nigeria), and we found no studies in 36 of the 47 WHO-defined African countries. While more research is needed on translations of interventions across low-resource settings, we urgently need ground-work research in local contexts. Surprisingly, the general population detained in prisons was poorly represented in our sample, as prison studies were concentrated around people with particular psychiatric or forensic variables such as type of crime or trial status.

Our meta-analysis of prevalence studies revealed high pooled prevalence of mental disorders and substance use among PDJS in Africa, which underscores the urgency of addressing the mental health of detained people in Africa within the global mental health movement. However, the studies were heterogeneous. While we attempted to explore and explain the heterogeneity using subgroup analysis, nearly all subgroups were also heterogeneous. The most valid finding of the meta-analysis is that we have statistically shown that the studies were conducted on distinct populations. This is not surprising given that the populations are from across Africa and are detained in a variety of different types of institutions and under distinct penal policies. Accordingly, it is important to interpret the meta-analysis findings cautiously as the heterogeneity may limit the validity of providing a single point estimate for distinct populations. Notably, the level of heterogeneity we found was consistent with prior systematic reviews on the prevalence of mental disorders in prison settings [2]. All of this underscores the need for more research on mental health in prison settings and better standardization of the tools used to assess mental and substance use disorders.

While recognizing the heterogeneity, prevalence estimates are consistently higher than those estimated in a prior systematic review on mental illness in prison, which found a 3.6% prevalence of psychosis among men and 3.9% in women, and a 10.2% prevalence of major depression in men and 14.1% in women [2]. This prior review, however, included only one study from Africa, where resources for psychiatric care are particularly limited, and it did not include forensic wards. When we examined the prevalence of psychotic disorders in prisons alone (forensic psychiatry units excluded), the prevalence of psychotic disorders (1%; 95% CI 0.00–0.02) was much more similar to the prior review, but the prevalence of mood disorders (33%; 95% CI 0.20–0.46) remained higher. Although the prior review estimated the prevalence of major depression alone while this review includes bipolar disorder, almost all studies included in our estimate of mood disorders in prisons measured depression exclusively, and there was low prevalence of bipolar disorder. The prevalence of substance use disorder (35%; 95% CI 0.25–0.44) was similarly high as observed in a prior international systematic review on substance use and dependence in prison, which found prevalence for alcohol use and dependence ranged from 18 to 30% among men and 10 to 24% among women, while prevalence of drug abuse and dependence varied from 10 to 48% in male prisoners and 30 to 60% in female prisoners [4]. There were too few women included in our study results to warrant meta-analysis by gender; however, the gender differences observed in prior research highlight the need for intentional data collection on gender in future work.

Subgroup analysis highlighted that there are differences in prevalence between institutions, with higher prevalence of mood disorders in prisons, higher prevalence of psychotic disorders in forensic institutions, and very low prevalence of psychotic disorders in prisons. This finding suggests that people with severe psychotic illnesses are being tracked out of prisons and into forensic units, which theoretically provide more psychiatric treatment. Interestingly, however, the prevalence of any sort of mental disorder in forensic units was less than 100%, indicating that people without psychiatric diagnoses are being detained in these units in some settings. Additionally, there was relatively high prevalence of substance use across facilities, highlighting the importance of considering substance use when developing interventions in all settings of detention. The prevalence of any mental disorder in youth institutions had particularly high variability, perhaps because of the small number of studies and wide variety in reasons for youth detention. The findings of the meta-analysis, however, must be interpreted cautiously because the samples are highly heterogeneous, likely because these samples are drawn from different countries, institutions, and cultural contexts, and the outcomes are measured in a variety of ways with tools that may not be adequately validated in their settings. The heterogeneity is expected in a field with such paucity of data and is consistent with other meta-analyses of mental disorders in prisons [2].

Systematic reviews have the potential to include, and even elevate, ethically questionable studies due to the nature of an exhaustive search [31, 63]. This may be particularly problematic for research involving vulnerable populations such as PDJS. We chose not to exclude any studies based on their ethical practices because excluding studies potentially detracts from knowledge that should eventually be used to help this vulnerable population. Thirty-five percent of studies failed to document ethics committee approval or informed consent procedures. Not all journals require ethics reporting, so our results do not necessarily indicate whether ethics procedures actually occurred, but raise concerns. The use of ethics assessment protocols in systematic reviews [31] and greater standardization for ethics reporting across journals could help ensure that all relevant ethical procedures are described.

The three effective interventions studies identified in the review may provide a starting point for future development of interventions and could be adapted for other settings. For instance, two interventions—one for depression [42], one for substance dependence [43]—used cognitive behavioral therapy approaches in group-focused sessions held twice a week. This group-focused, cognitive behavioral design could be adapted in future psychosocial interventions. The other intervention, a RCT for neuroleptics used to treat schizophrenia symptoms [44], highlights the strengths of pharmacological interventions for severe mental illness.

## Implications for health workers and policymakers

First, we were surprised at how many studies included people who had not been convicted. Considerable work at all levels is needed to ensure that people with mental illness have rapid access to trial and are not detained without due process. Second, the high prevalence of mental disorders demonstrated across all settings of the justice system highlights the need for all staff members to receive mental health training. Likewise, those working in mental health should receive training on managing the needs of people who have been involved with the justice system. However, interventions must be coordinated and services designed to support individuals as they move across health, justice, and social systems. For instance, Munetz and Griffin propose the sequential intercept model for diversion of people with mental illness from the justice system in which various points of contact with the justice system become opportunities for connecting individuals to services; i.e. interceptions to prevent further justice involvement [3, 64]. Third, the prevalence of substance use disorders was particularly high. Systematic reviews and RCTs from HICs highlight the importance of medication-assisted interventions, which combine psychological and pharmacological treatment, for substance use disorders in PDJS [3, 65, 66].

## Directions for future research

First, the studies included have made it clear that there is high psychiatric prevalence among PDJS, but little development or testing of interventions to address the large mental health need. Contextual research on each system of detention and subpopulation, including youth, and older participants, trial or charge status, gender, and diagnosis, is essential to inform such interventions. In addition to providing mental health services within institutions of detention, the high prevalence of mental illness compared to the general populations suggests a need for research on supportive diversions of individuals with mental illness from the justice system to best connect individuals with care. Second, we have focused on the academic literature, but there should also be systematic and comprehensive data collection and analysis of institutional and governmental documents surrounding the mental health of PDJS in Africa. Third, there is need for longitudinal studies that follow participants to community re-entry, especially investigating transitions to community health care and long-term health and recidivism outcomes [67, 68]. Fourth, only five studies in this review collected qualitative data from PDJS themselves. Studies must do more to include service user voices. Fifth, prisons, youth institutions, and forensic psychiatry settings must be investigated both discretely and as facilities that feed into each other: each settings’ population has specific needs, but there may be revolving door effect for forensic psychiatric units and prisons, as described in Zimbabwe [49]. Sixth, economics research was not present in our results, but is needed to measure the societal costs of lack of treatment, the cost-effectiveness of treatments, and the potential cost-savings provided by interventions and diversions.

## Limitations

First, a major limitation for this review was the heterogeneity of studies and overall paucity of high quality literature. We chose broader definitions of detention and mental illness to provide a comprehensive perspective on this understudied area and better define the state of the field in Africa. However, because results were more variable than expected, we were unable to analyze specific aspects or subpopulations (interventions, forensic units, youth institutions, policy) in as much depth as we would have liked. We aim to delve deeper in the future, producing discrete publications on forensic hospitals, youth institutions, conditions of detention, but reported all results of this paper collectively in accordance with PRISMA guidelines [23], and to avoid legal, ethical, and methodological issues that arise from post hoc changes to the protocol and attempts to publish slices or versions of data that have been published previously [69]. This heterogeneity in included studies makes it challenging to point to singular conclusions from the data. However, the heterogeneity of study settings is a strength since exhaustive inclusion allows us to speak to the state of the issue in Africa at large and gives readers a more comprehensive overview of key areas for future work. This approach calls attention to the systems-wide nature of detention of people with mental illness, whereas prior reviews have excluded non-prison populations with high psychiatric prevalence that are similarly detained by the state. Second, because we added the meta-analysis post hoc, it was not included in our original protocol. We did not select studies with the aim of having a homogenous set of outcomes or study designs to facilitate meta-analysis. Additionally, the disease categories we used to group analysis were generated inductively based on the included studies. They were broad and likely introduced additional heterogeneity into the groups. However, we believe that meta-analysis provides one additional way for readers to access and understand our data and triangulates the results of the narrative review, which were consistent with the meta-analysis findings. Third, we discovered eight studies during the backward search: a signal that perhaps the original search strategy was imperfect. Many of these studies were from small journals that were not indexed in the major databases that we had originally chosen to search. While we could have iteratively changed the search strategy to broaden the list of databases we searched, we chose to search only large databases of established quality and to adhere to our study protocol. Finally, a unifying theme throughout our review and in evaluating our limitations has been a data quality issue, as many studies had high risk of bias or reported so little about their methods that it was difficult to assess risk of bias.

## Conclusion

This review has identified key areas that require further research, and demonstrated need for more standardized methods and ethics reporting. It has confirmed the high prevalence of mental illness among PDJS in Africa, but revealed an absence of setting diversity or diversity of study types, and revealed key populations under-researched or missing from the literature. Though the need for bio-medically focused interventions is clear from this high psychiatric prevalence, we look forward to a future in which prevention approaches and social interventions are prioritized. Social factors of stress, poverty, and discrimination may disproportionately affect people that become detained and contribute to poor mental health. Future mental health research must take on a systems-wide perspective involving both the health and justice sectors, and investigate both clinical and contextual social variables. This approach will guide interventions for coordinated service development, and better align policy with the aim of the Sustainable Development Goals’ to leave no one behind in achieving equitable, universal health coverage [70, 71].

## Additional file

**Additional file 1.** Appendices S1–S11.
