# Supplementary material for: Mental health of people detained within the justice system in Africa: systematic review and meta-analysis
Source: Int J Ment Health Syst. 2019 May 6;13:31. doi: 10.1186/s13033-019-0273-z (PMC6501291; doi:10.1186/s13033-019-0273-z)
Supplement: Supplementary file 1 — Additional file 1. Appendices S1–S11. [file 13033_2019_273_MOESM1_ESM.docx]

**Appendix**

**S1. Preferred Reporting Items for Systematic Review and Meta-analyses (PRISMA) Checklist**

| **Section/topic** | **#** | **Checklist item** | **Reported on page #** |
| --- | --- | --- | --- |
| **TITLE** | | |  |
| Title | 1 | Identify the report as a systematic review, meta-analysis, or both. | 1 |
| **ABSTRACT** | | |  |
| Structured summary | 2 | Provide a structured summary including, as applicable: background; objectives; data sources; study eligibility criteria, participants, and interventions; study appraisal and synthesis methods; results; limitations; conclusions and implications of key findings; systematic review registration number. | 2, 5 |
| **INTRODUCTION** | | |  |
| Rationale | 3 | Describe the rationale for the review in the context of what is already known. | 4 |
| Objectives | 4 | Provide an explicit statement of questions being addressed with reference to participants, interventions, comparisons, outcomes, and study design (PICOS). | 4-5 |
| **METHODS** | | |  |
| Protocol and registration | 5 | Indicate if a review protocol exists, if and where it can be accessed (e.g., Web address), and, if available, provide registration information including registration number. | 5 |
| Eligibility criteria | 6 | Specify study characteristics (e.g., PICOS, length of follow-up) and report characteristics (e.g., years considered, language, publication status) used as criteria for eligibility, giving rationale. | 5 |
| Information sources | 7 | Describe all information sources (e.g., databases with dates of coverage, contact with study authors to identify additional studies) in the search and date last searched. | 5-6 |
| Search | 8 | Present full electronic search strategy for at least one database, including any limits used, such that it could be repeated. | Appendix S3 |
| Study selection | 9 | State the process for selecting studies (i.e., screening, eligibility, included in systematic review, and, if applicable, included in the meta-analysis). | 5-7 |
| Data collection process | 10 | Describe method of data extraction from reports (e.g., piloted forms, independently, in duplicate) and any processes for obtaining and confirming data from investigators. | 6-7 |
| Data items | 11 | List and define all variables for which data were sought (e.g., PICOS, funding sources) and any assumptions and simplifications made. | 5-7, S4 |
| Risk of bias in individual studies | 12 | Describe methods used for assessing risk of bias of individual studies (including specification of whether this was done at the study or outcome level), and how this information is to be used in any data synthesis. | 6 |
| Summary measures | 13 | State the principal summary measures (e.g., risk ratio, difference in means). | 6-7 |
| Synthesis of results | 14 | Describe the methods of handling data and combining results of studies, if done, including measures of consistency (e.g., I^2^) for each meta-analysis. | 6-7 |
| Risk of bias across studies | 15 | Specify any assessment of risk of bias that may affect the cumulative evidence (e.g., publication bias, selective reporting within studies). | 6 |
| Additional analyses | 16 | Describe methods of additional analyses (e.g., sensitivity or subgroup analyses, meta-regression), if done, indicating which were pre-specified. | 7 |
| **RESULTS** |  |  |  |
| Study selection | 17 | Give numbers of studies screened, assessed for eligibility, and included in the review, with reasons for exclusions at each stage, ideally with a flow diagram. | 8, Figure 1 |
| Study characteristics | 18 | For each study, present characteristics for which data were extracted (e.g., study size, PICOS, follow-up period) and provide the citations. | 8-10, Tables 2-3 (p. 22-45) |
| Risk of bias within studies | 19 | Present data on risk of bias of each study and, if available, any outcome level assessment (see item 12). | 9, S5; Tables 2-3 (p. 22-45), Appendix S5 |
| Results of individual studies | 20 | For all outcomes considered (benefits or harms), present, for each study: (a) simple summary data for each intervention group (b) effect estimates and confidence intervals, ideally with a forest plot. | Tables 2-3 (p. 22-45), S8, S10, S11 |
| Synthesis of results | 21 | Present results of each meta-analysis done, including confidence intervals and measures of consistency. | 10-12 |
| Risk of bias across studies | 22 | Present results of any assessment of risk of bias across studies (see Item 15). | 9, Appendix S5 |
| Additional analysis | 23 | Give results of additional analyses, if done (e.g., sensitivity or subgroup analyses, meta-regression [see Item 16]). | 10-12 |
| **DISCUSSION** |  |  |  |
| Summary of evidence | 24 | Summarize the main findings including the strength of evidence for each main outcome; consider their relevance to key groups (e.g., healthcare providers, users, and policy makers). | 14-18 |
| Limitations | 25 | Discuss limitations at study and outcome level (e.g., risk of bias), and at review-level (e.g., incomplete retrieval of identified research, reporting bias). | 18 |
| Conclusions | 26 | Provide a general interpretation of the results in the context of other evidence, and implications for future research. | 19 |
| **FUNDING** |  |  |  |
| Funding | 27 | Describe sources of funding for the systematic review and other support (e.g., supply of data); role of funders for the systematic review. | 20-21 |

******Page numbers may change during publication*

**S2. Meta-Analysis of Observational Studies in Epidemiology (MOOSE) Checklist**

| **#** | **Section** | **Reported on page #** | **Notes** |
| --- | --- | --- | --- |
| **Reporting of background should include** | | | |
| 1 | Problem definition | 4-5 |  |
| 2 | Hypothesis statement | N/A | This review’s aim was to provide a broad overview of the existing literature and not to test a specific hypothesis. |
| 3 | Description of study outcomes | 6-7 | Prevalence |
| 4 | Type of exposure or intervention used | 5-6, 10 | Detention within a prison, youth institution, or forensic ward |
| 5 | Type of study designs used | 5-6, 10-11 | Prevalence |
| 6 | Study population | 5 |  |
| **Reporting of search strategy should include** | | | |
| 7 | Qualifications of searchers | 6 |  |
| 8 | Search strategy, including time period included in the synthesis and key words | 5-6, Appendix S3 |  |
| 9 | Effort to include all available studies, including contact with authors | 5-6 | 6: backward search, author contact |
| 10 | Databases and registries searched | 5 |  |
| 11 | Search software used, name and version, including special features used (e.g., explosion) | 5 |  |
| 12 | Use of hand searching (e.g., reference lists of obtained articles) | 6 | 6: backward search |
| 13 | List of citations located and those excluded, including justification | Figure 1, Appendix S3 | Reasons for exclusion shown in Figure 1 |
| 14 | Method of addressing articles published in languages other than English | 5 | Included studies published in English or French, the main languages of research on the African continent |
| 15 | Method of handling abstracts and unpublished studies | 5 |  |
| 16 | Description of any contact with authors | 6 |  |
| **Reporting of methods should include** | | | |
| 17 | Description of relevance or appropriateness of studies assembled for assessing the hypothesis to be tested | 5-6 | Selection criteria, risk of bias assessment |
| 18 | Rationale for the selection and coding of data (e.g., sound clinical principles or convenience) | 6-7 | Data extraction |
| 19 | Documentation of how data were classified and coded (e.g., multiple raters, blinding, and interrater reliability) | 6-7 | Data extraction |
| 20 | Assessment of confounding (e.g., comparability of cases and controls in studies where appropriate) | N/A |  |
| 21 | Assessment of study quality, including blinding of quality assessors; stratification or regression on possible predictors of study results | 6 | Risk of bias assessment |
| 22 | Assessment of heterogeneity | 7 |  |
| 23 | Description of statistical methods (e.g., complete description of fixed or random effects models, justification of whether the chosen models account for predictors of study results, dose-response models, or cumulative meta-analysis) in sufficient detail to be replicated | 7 |  |
| 24 | Provision of appropriate tables and graphics | Figure 1, Table S5 |  |
| **Reporting of results should include** | | | |
| 25 | Graphic summarizing individual study estimates and overall estimate | Figures 2-5 |  |
| 26 | Table giving descriptive information for each study included | Table 2 |  |
| 27 | Results of sensitivity testing (e.g., subgroup analysis) | Tables 5, S6, S7 |  |
| 28 | Indication of statistical uncertainty of findings | Tables 4-5, Figures 2-5 |  |
| **Reporting of results should include** | | | |
| 29 | Quantitative assessment of bias (e.g., publication bias) | Table S5, Figures 2-5 |  |
| 30 | Justification for exclusion (e.g., exclusion of non–English-language citations) | 8, Figure 1 |  |
| 31 | Assessment of quality of included studies | 9, Table S5 |  |
| **Reporting of conclusions should include** | | | |
| 32 | Consideration of alternative explanations for observed results | 14-16 |  |
| 33 | Generalization of the conclusions (i.e., appropriate for the data presented and within the domain of the literature review) | 16-19 |  |
| 34 | Guidelines for future research | 17 |  |
| 35 | Disclosure of funding source | 20-21 |  |

******Page numbers may change during publication*

**S3. Search Terms by Database**

Database: PubMed (NCBI)

Date: 16 November 2017

474 Records

("Prisons"[Mesh] OR "Prisoners"[Mesh:NoExp] OR prison*[tiab] OR imprison*[tiab] OR jail*[tiab] OR incarcerat*[tiab] OR correctional facilit*[tiab] OR offender*[tiab] OR criminal*[tiab] OR inmate*[tiab] OR probation*[tiab] OR remand*[tiab] OR felon*[tiab] OR penitentiar*[tiab] OR detainee*[tiab])

AND

("Mental Health"[Mesh] OR "Mental Disorders"[Mesh] OR "Mental Health Services"[Mesh] OR "Suicide"[Mesh] OR mental health[tiab] OR mental illness*[tiab] OR mental disorder*[tiab] OR psychiatric[tiab] OR psychiatry[tiab] OR psychosomatic[tiab] OR depression[tiab] OR depressive[tiab] OR bipolar[tiab] OR mood disorder*[tiab] OR affective disorder*[tiab] OR anxiety[tiab] OR panic disorder*[tiab] OR obsessive compulsive[tiab] OR ocd[tiab] OR stress disorder*[tiab] OR stress symptom*[tiab] OR stress syndrome*[tiab] OR traumatic stress*[tiab] OR posttraumatic stress*[tiab] OR ptsd[tiab] OR substance use[tiab] OR substance abuse[tiab] OR drug use*[tiab] OR drug abuse*[tiab] OR drug misuse*[tiab] OR alcohol*[tiab] OR drinking[tiab] OR opioid*[tiab] OR opiate*[tiab] OR narcotic*[tiab] OR heroin[tiab] OR morphine[tiab] OR opium[tiab] OR marijuana[tiab] OR cannabis[tiab] OR hashish[tiab] OR amphetamine*[tiab] OR glue[tiab] OR inhalant*[tiab] OR schizophren*[tiab] OR psychosis[tiab] OR psychotic[tiab] OR insane[tiab] OR insanity[tiab] OR self harm*[tiab] OR suicid*[tiab])

AND

("Developing Countries"[mesh] OR developing countr*[tiab] OR developing nation*[tiab] OR less developed countr*[tiab] OR less developed nation*[tiab] OR third world nation*[tiab] OR third world countr*[tiab] OR under developed nation*[tiab] OR underdeveloped nation*[tiab] OR under developed countr*[tiab] OR underdeveloped nation*[tiab] OR low income countr*[tiab] OR low income nation*[tiab] OR poor countr*[tiab] OR poor nation*[tiab] OR lmic[tiab] OR lmics[tiab] OR "Africa"[mesh] OR africa[tiab] OR east africa*[tiab] OR eastern africa*[tiab] OR north africa*[tiab] OR northern africa*[tiab] OR central africa*[tiab] OR west africa*[tiab] OR western africa*[tiab] OR south africa*[tiab] OR southern africa*[tiab] OR maghreb[tiab] OR sahara*[tiab] OR subsahara*[tiab] OR sahel[tiab] OR Algeria*[tiab] OR Angola*[tiab] OR Benin*[tiab] OR Burkina Faso[tiab] OR Cameroon*[tiab] OR Cape Verde*[tiab] OR Chad*[tiab] OR Guinea*[tiab] OR Gabon*[tiab] OR Gambia*[tiab] OR Ghana*[tiab] OR Liberia*[tiab] OR Madagascar*[tiab] OR Mali[tiab] OR malian[tiab] OR malians[tiab] OR Mauritania*[tiab] OR Mauriti*[tiab] OR Niger*[tiab] OR Nigeria*[tiab] OR Sao Tome and Principe*[tiab] OR Senegal*[tiab] OR Seychelles*[tiab] OR Sierra Leone*[tiab] OR Togo*[tiab] OR Botswana*[tiab] OR Burundi*[tiab] OR Congo*[tiab] OR CÙte d'Ivoire[tiab] OR ivory coast[tiab] OR ivorian[tiab] OR Eritrea*[tiab] OR Ethiopia*[tiab] OR Kenya*[tiab] OR Lesotho*[tiab] OR Malawi*[tiab] OR Mozambique*[tiab] OR Namibia*[tiab] OR Rwanda*[tiab] OR Swaziland*[tiab] OR Uganda*[tiab] OR Tanzania*[tiab] OR Zambia*[tiab] OR Zimbabwe[tiab] OR Algeria*[ad] OR Angola*[ad] OR Benin*[ad] OR Burkina Faso[ad] OR Cameroon*[ad] OR Cape Verde*[ad] OR Chad*[ad] OR Guinea*[ad] OR Gabon*[ad] OR Gambia*[ad] OR Ghana*[ad] OR Liberia*[ad] OR Madagascar*[ad] OR Mali[ad] OR Mauritania*[ad] OR Mauriti*[ad] OR Niger*[ad] OR Nigeria*[ad] OR Sao Tome and Principe*[ad] OR Senegal*[ad] OR Seychelles*[ad] OR Sierra Leone*[ad] OR Togo*[ad] OR Botswana*[ad] OR Burundi*[ad] OR Central African Republic*[ad] OR Congo*[ad] OR CÙte d'Ivoire[ad] OR ivory coast[ad] OR ivorian[ad] OR Eritrea*[ad] OR Ethiopia*[ad] OR Kenya*[ad] OR Lesotho*[ad] OR Malawi*[ad] OR Mozambique*[ad] OR Namibia*[ad] OR Rwanda*[ad] OR Swaziland*[ad] OR Uganda*[ad] OR Tanzania*[ad] OR Zambia*[ad] OR Zimbabwe[ad])

Embase (Elsevier)

Date: 16 November 2017

540 Records

('prison'/exp OR 'prisoner'/de OR prison*:ab,ti OR imprison*:ab,ti OR jail*:ab,ti OR incarcerat*:ab,ti OR 'correctional facility':ab,ti OR 'correctional facilities':ab,ti OR offender*:ab,ti OR criminal*:ab,ti OR inmate*:ab,ti OR probation*:ab,ti OR remand*:ab,ti OR felon*:ab,ti OR penitentiar*:ab,ti OR detainee*:ab,ti)

AND

('mental health'/exp OR 'mental health service'/exp OR 'mental disease'/exp OR 'suicide'/exp OR (mental NEXT/1 (health OR illness* OR disorder*)):ab,ti OR psychiatric:ab,ti OR psychiatry:ab,ti OR psychosomatic:ab,ti OR depression:ab,ti OR depressive:ab,ti OR bipolar:ab,ti OR ((mood OR affective OR panic OR stress) NEXT/1 disorder*):ab,ti OR anxiety:ab,ti OR 'obsessive compulsive':ab,ti OR ocd:ab,ti OR (stress NEXT/1 (symptom* OR syndrome*)):ab,ti OR ((traumatic OR posttraumatic) NEXT/1 stress):ab,ti OR ptsd:ab,ti OR ((substance OR drug OR drugs) NEAR/1 (use* OR abuse* OR misuse*)):ab,ti OR alcohol*:ab,ti OR drinking:ab,ti OR opioid*:ab,ti OR opiate*:ab,ti OR narcotic*:ab,ti OR heroin:ab,ti OR morphine:ab,ti OR opium:ab,ti OR marijuana:ab,ti OR cannabis:ab,ti OR hashish:ab,ti OR amphetamine*:ab,ti OR glue:ab,ti OR inhalant*:ab,ti OR schizophren*:ab,ti OR psychosis:ab,ti OR psychotic:ab,ti OR insane:ab,ti OR insanity:ab,ti OR (self NEXT/1 harm*):ab,ti OR suicid*:ab,ti)

AND

('developing country'/exp OR ((developing OR 'less developed' OR 'third world' OR 'under developed' OR 'middle income' OR 'low income' OR underserved OR 'under served' OR deprived OR poor*) NEAR/1 (count* OR nation* OR state* OR population*)):ab,ad,ti OR lmic:ab,ad,ti OR lmics:ab,ad,ti OR 'Africa'/exp OR africa:ab,ti OR ((east OR eastern OR north OR northern OR central OR western OR south OR southern) NEXT/1 africa*):ab,ad,ti OR maghreb:ab,ti OR sahara*:ab,ti OR subsahara*:ab,ti OR sahel:ab,ad,ti OR Algeria*:ab,ad,ti OR Angola*:ab,ad,ti OR Benin*:ab,ad,ti OR 'Burkina Faso':ab,ad,ti OR Cameroon*:ab,ad,ti OR (Cape NEXT/1 Verde*):ab,ad,ti OR Chad*:ab,ad,ti OR Guinea*:ab,ad,ti OR Gabon*:ab,ad,ti OR Gambia*:ab,ad,ti OR Ghana*:ab,ad,ti OR Liberia*:ab,ad,ti OR Madagascar*:ab,ad,ti OR Mali:ab,ad,ti OR malian:ab,ad,ti OR malians:ab,ad,ti OR Mauritania*:ab,ad,ti OR Mauriti*:ab,ad,ti OR Niger*:ab,ad,ti OR Nigeria*:ab,ad,ti OR 'Sao Tome':ab,ad,ti OR Principe*:ab,ad,ti OR Senegal*:ab,ad,ti OR Seychelles*:ab,ad,ti OR 'Sierra Leone':ab,ad,ti OR Togo*:ab,ad,ti OR Botswana*:ab,ad,ti OR Burundi*:ab,ad,ti OR Congo*:ab,ad,ti OR 'CÙte d Ivoire':ab,ad,ti OR 'ivory coast':ab,ad,ti OR ivorian:ab,ad,ti OR Eritrea*:ab,ad,ti OR Ethiopia*:ab,ad,ti OR Kenya*:ab,ad,ti OR Lesotho*:ab,ad,ti OR Malawi*:ab,ad,ti OR Mozambique*:ab,ad,ti OR Namibia*:ab,ad,ti OR Rwanda*:ab,ad,ti OR Swaziland*:ab,ad,ti OR Uganda*:ab,ad,ti OR Tanzania*:ab,ad,ti OR Zambia*:ab,ad,ti OR Zimbabwe:ab,ad,ti)

PsycINFO (EBSCO)

Date: 16 November 2017

386 Records

(DE ("Correctional Institutions" OR "Prisons" OR "Prisoners" OR "Incarceration" OR "Maximum Security Facilities") OR TI (prison* OR imprison* OR jail* OR incarcerat* OR "correctional facility" OR "correctional facilities" OR offender* OR criminal* OR inmate* OR probation* OR remand* OR felon* OR penitentiar* OR detainee*) OR AB (prison* OR imprison* OR jail* OR incarcerat* OR "correctional facility" OR "correctional facilities" OR offender* OR criminal* OR inmate* OR probation* OR remand* OR felon* OR penitentiar* OR detainee*))

AND

(DE ("Mental Health" OR "Mental Health Services" OR "Mental Disorders" OR "Bipolar Disorder" OR "Major Depression" OR "Mania" OR "Acute Stress Disorder" OR "Obsessive Compulsive Disorder" OR "Panic Disorder" OR "Post-Traumatic Stress" OR "Posttraumatic Stress Disorder" OR "Anxiety Disorders" OR "Generalized Anxiety Disorder" OR "Substance Use Disorder" OR "Drug Abuse" OR "Drug Addiction" OR "Drug Dependency" OR "Alcohol Abuse" OR "Inhalant Abuse" OR "Polydrug Abuse" OR "Suicide") OR TI ((mental W1 (health OR illness* OR disorder*)) OR psychiatric OR psychiatry OR psychosomatic OR depression OR depressive OR bipolar OR ((mood OR affective OR panic OR stress) W1 disorder*) OR anxiety OR "obsessive compulsive" OR ocd OR (stress W1 (symptom* OR syndrome*)) OR ((traumatic OR posttraumatic) W1 stress) OR ptsd OR ((substance OR drug OR drugs) N1 (use* OR abuse* OR misuse*)) OR alcohol* OR drinking OR opioid* OR opiate* OR narcotic* OR heroin OR morphine OR opium OR marijuana OR cannabis OR hashish OR amphetamine* OR glue OR inhalant* OR schizophren* OR psychosis OR psychotic OR insane OR insanity OR "self harm*" OR suicid*) OR AB ((mental W1 (health OR illness* OR disorder*)) OR psychiatric OR psychiatry OR psychosomatic OR depression OR depressive OR bipolar OR ((mood OR affective OR panic OR stress) W1 disorder*) OR anxiety OR "obsessive compulsive" OR ocd OR (stress W1 (symptom* OR syndrome*)) OR ((traumatic OR posttraumatic) W1 stress) OR ptsd OR ((substance OR drug OR drugs) N1 (use* OR abuse* OR misuse*)) OR alcohol* OR drinking OR opioid* OR opiate* OR narcotic* OR heroin OR morphine OR opium OR marijuana OR cannabis OR hashish OR amphetamine* OR glue OR inhalant* OR schizophren* OR psychosis OR psychotic OR insane OR insanity OR "self harm*" OR suicid*))

AND

DE "Developing Countries" OR TI (((developing OR "less developed" OR "third world" OR "under developed" OR "middle income" OR "low income" OR underserved OR "under served" OR deprived OR poor*) N1 (count* OR nation*)) OR lmic OR lmics OR africa OR ((east OR eastern OR north OR northern OR central OR western OR south OR southern) W1 africa*) OR maghreb OR sahara* OR subsahara* OR sahel OR Algeria* OR Angola* OR Benin* OR "Burkina Faso" OR Cameroon* OR "Cape Verd*" OR Chad* OR Guinea* OR Gabon* OR Gambia* OR Ghana* OR Liberia* OR Madagascar* OR Mali OR malian OR malians OR Mauritania* OR Mauriti* OR Niger* OR Nigeria* OR "Sao Tome" OR Principe* OR Senegal* OR Seychelles* OR "Sierra Leone" OR Togo* OR Botswana* OR Burundi* OR Congo* OR "CÙte d Ivoire" OR "ivory coast" OR ivorian OR Eritrea* OR Ethiopia* OR Kenya* OR Lesotho* OR Malawi* OR Mozambique* OR Namibia* OR Rwanda* OR Swaziland* OR Uganda* OR Tanzania* OR Zambia* OR Zimbabw*) OR AB (((developing OR "less developed" OR "third world" OR "under developed" OR "middle income" OR "low income" OR underserved OR "under served" OR deprived OR poor*) N1 (count* OR nation*)) OR lmic OR lmics OR africa OR ((east OR eastern OR north OR northern OR central OR western OR south OR southern) W1 africa*) OR maghreb OR sahara* OR subsahara* OR sahel OR Algeria* OR Angola* OR Benin* OR "Burkina Faso" OR Cameroon* OR "Cape Verd*" OR Chad* OR Guinea* OR Gabon* OR Gambia* OR Ghana* OR Liberia* OR Madagascar* OR Mali OR malian OR malians OR Mauritania* OR Mauriti* OR Niger* OR Nigeria* OR "Sao Tome" OR Principe* OR Senegal* OR Seychelles* OR "Sierra Leone" OR Togo* OR Botswana* OR Burundi* OR Congo* OR "CÙte d Ivoire" OR "ivory coast" OR ivorian OR Eritrea* OR Ethiopia* OR Kenya* OR Lesotho* OR Malawi* OR Mozambique* OR Namibia* OR Rwanda* OR Swaziland* OR Uganda* OR Tanzania* OR Zambia* OR Zimbabwe) OR AF (Algeria* OR Angola* OR Benin* OR "Burkina Faso" OR Cameroon* OR "Cape Verd*" OR Chad* OR Guinea* OR Gabon* OR Gambia* OR Ghana* OR Liberia* OR Madagascar* OR Mali OR malian OR malians OR Mauritania* OR Mauriti* OR Niger* OR Nigeria* OR "Sao Tome" OR Principe* OR Senegal* OR Seychelles* OR "Sierra Leone" OR Togo* OR Botswana* OR Burundi* OR Congo* OR "CÙte d Ivoire" OR "ivory coast" OR ivorian OR Eritrea* OR Ethiopia* OR Kenya* OR Lesotho* OR Malawi* OR Mozambique* OR Namibia* OR Rwanda* OR Swaziland* OR Uganda* OR Tanzania* OR Zambia* OR Zimbabwe OR "South Africa")

Web of Science (Clarivate Analytics)

Date: 16 November 2017

499 Records

TS=("prison*" OR "imprison*" OR "jail*" OR "incarcerat*" OR "correctional facility" OR "correctional facilities" OR "offender*" OR "criminal*" OR "inmate*" OR "probation*" OR "remand*" OR "felon*" OR "penitentiar*" OR "detainee*")

AND

TS=(("mental" NEAR/1 ("health" OR "illness*" OR "disorder*")) OR "psychiatric" OR "psychiatry" OR "psychosomatic" OR "depression" OR "depressive" OR "bipolar" OR (("mood" OR "affective" OR "panic" OR "stress") NEAR/1 "disorder*") OR "anxiety" OR "obsessive compulsive" OR "ocd" OR ("stress" NEAR/1 ("symptom*" OR "syndrome*")) OR (("traumatic" OR "posttraumatic") NEAR/1 "stress") OR "ptsd" OR (("substance" OR "drug" OR "drugs") NEAR/1 ("use*" OR "abuse*" OR "misuse*")) OR "alcohol*" OR "drinking" OR "opioid*" OR "opiate*" OR "narcotic*" OR "heroin" OR "morphine" OR "opium" OR "marijuana" OR "cannabis" OR "hashish" OR "amphetamine*" OR "glue" OR "inhalant*" OR "schizophren*" OR "psychosis" OR "psychotic" OR "insane" OR "insanity" OR "self harm*" OR "suicid*")

AND

(TS=((("developing" OR "less developed" OR "third world" OR "under developed" OR "middle income" OR "low income" OR "underserved" OR "under served" OR "deprived" OR "poor*") NEAR/1 ("count*" OR "nation*" OR "state*" OR "population*")) OR "lmic" OR "lmics" OR "africa" OR (("east" OR "eastern" OR "north" OR "northern" OR "central" OR "western" OR "south" OR "southern") NEAR/1 "africa*") OR "maghreb" OR "sahara*" OR "subsahara*" OR "sahel" OR "Algeria*" OR "Angola*" OR "Benin*" OR "Burkina Faso" OR "Cameroon*" OR "Cape Verde*" OR "Chad*" OR "Guinea*" OR "Gabon*" OR "Gambia*" OR "Ghana*" OR "Liberia*" OR "Madagascar*" OR "Mali" OR "malian" OR "malians" OR "Mauritania*" OR "Mauriti*" OR "Niger*" OR "Nigeria*" OR "Sao Tome" OR "Principe*" OR "Senegal*" OR "Seychelles*" OR "Sierra Leone" OR "Togo*" OR "Botswana*" OR "Burundi*" OR "Congo*" OR "CÙte d Ivoire" OR "ivory coast" OR "ivorian" OR "Eritrea*" OR "Ethiopia*" OR "Kenya*" OR "Lesotho*" OR "Malawi*" OR "Mozambique*" OR "Namibia*" OR "Rwanda*" OR "Swaziland*" OR "Uganda*" OR "Tanzania*" OR "Zambia*" OR "Zimbabwe") OR AD=("Algeria*" OR "Angola*" OR "Benin*" OR "Burkina Faso" OR "Cameroon*" OR "Cape Verde*" OR "Chad*" OR "Guinea*" OR "Gabon*" OR "Gambia*" OR "Ghana*" OR "Liberia*" OR "Madagascar*" OR "Mali" OR "malian" OR "malians" OR "Mauritania*" OR "Mauriti*" OR "Niger*" OR "Nigeria*" OR "Sao Tome" OR "Principe*" OR "Senegal*" OR "Seychelles*" OR "Sierra Leone" OR "Togo*" OR "Botswana*" OR "Burundi*" OR "Congo*" OR "CÙte d Ivoire" OR "ivory coast" OR "ivorian" OR "Eritrea*" OR "Ethiopia*" OR "Kenya*" OR "Lesotho*" OR "Malawi*" OR "Mozambique*" OR "Namibia*" OR "Rwanda*" OR "Swaziland*" OR "Uganda*" OR "Tanzania*" OR "Zambia*" OR "Zimbabwe"))

African Index Medicus (WHO Global Index Medicus)

Date: 16 November 2017

12 Records

(prison* OR imprison* OR jail* OR incarcerat* OR "correctional facility" OR "correctional facilities" OR offender* OR criminal* OR inmate* OR probation* OR remand* OR felon* OR penitentiar* OR detainee*)

AND

("mental health" OR "mental illness" OR "mental illnesses" OR "mental disorder" OR "mental disorders" OR psychiatric OR psychiatry OR psychosomatic OR depression OR depressive OR bipolar OR "mood disorder" OR "mood disorders" OR "affective disorder" OR "affective disorders" OR anxiety OR "panic disorder" OR "obsessive compulsive" OR ocd OR "stress disorder" OR "stress disorders" OR "stress symptom" OR "stress symptoms" OR "stress syndrome" OR "stress syndromes" OR "traumatic stress" OR "traumatic stresses" OR "posttraumatic stress" OR ptsd OR "substance use" OR "substance abuse" OR "drug use" OR "drug users" OR "drug abuse" OR "drug abusers" OR "drug misuse" OR alcohol* OR drinking OR opioid* OR opiate* OR narcotic* OR heroin OR morphine OR opium OR marijuana OR cannabis OR hashish OR amphetamine* OR glue OR inhalant* OR schizophren* OR psychosis OR psychotic OR insane OR insanity OR "self harm" OR "self harms" OR "self harming" OR suicid*)

**S4. Assumptions in meta-analysis**

In our meta-analysis, we made the following assumptions:

- Selection of data for inclusion
  - If a screening tool had both an overall score (for instance, presence or absence of mental illness) and diagnosis-specific sub-scales, we used only the overall score unless the paper specifically noted that a sub-scale was the primary outcome. As the validity of sub-scales differs considerably from overall scales, we did not believe that including sub-scales of screening tools was appropriate.
  - If a diagnostic tool had both an overall outcome (for instance, presence or absence of mental illness) and a specific diagnosis, we included both the overall outcome (under “mental ill health”) and the specific diagnoses, as these are the primary outcomes of a diagnostic tool without separate validity.
  - If both current and lifetime diagnostic data was reported, we used current prevalence rather than lifetime prevalence.
  - If multiple diagnoses fit within a single category (for instance, “alcohol dependence” and “drug dependence” were both assessed and separate prevalence was given), this was only included if the authors specified that patients got a single diagnosis (in which case the two prevalence could be added) or stated the total number with any type of substance dependence (in the case of this example).
  - We did not name the category “substance use disorders”, but “substance use” because not all studies were clear on whether the patients were using substances regularly or if they had a true disorder/dependence. If studies measured only intoxication at the time of a crime or at the time of the study, that was not counted in “substance use”, as we were seeking regular use and not one-time use.
  - If there were multiple disorders that fit into one of the diagnostic categories that we defined (for instance, a study measured the prevalence of alcohol use and of opioid use), and if it was unclear if the study had double counted some participants (those who had both alcohol use and opioid use), that prevalence was excluded, assuming it would not be accurate to sum the number of participants in each category because some would be counted twice.
- Analysis
  - There were a number of studies that measured the same diagnoses on the same sample of patients. Because some studies used different tools and measured different prevalence of the same disorder in the same population, the analysis was performed with only one of the samples (so as not to double the weight of this sample), but we performed a robustness analysis by substituting in the excluded study, and the outcomes were not significantly different. This happened in two cases, described below.
- Robustness analysis
  - Any mental illness:
    - Issa et al. or Yusef et al. instead of Adegunloye et al.: No significant difference
      - Random effects: 0.59 (0.49-0.69), 2501.06, 99.08%
      - Fixed effects: 0.55 (0.54-0.56)
  - Mood disorder
    - Uche and Princewell (2015) instead of Uche and Princewell (2016):
      - Random effects: 0.22 (0.16-0.28), 3367.01, 99.23
      - Fixed effects: 0.04 (0.04-0.05)
- Studies that were excluded because they were repeat samples (identical population, tool used, and prevalence outcome):
  - *Ajiboye et al. 2009 (same sample as Adegunloye)
  - *Olashore et al. 2017 (same as Olashore, 2016)
  - *Dachew et al. 2015 (same sample as Beyen)
  - *Armiya'u et al. 2013 " Prevalence of..." (same as Armiya'u et al. 2013 "A study of...")
  - *Atilola et al. 2017 "Status..." (same as Atilola et al. 2017 "Correlations...")

**S5. Methodologic Review of Prevalence Studies:** The Joanna Briggs Institute Prevalence Critical Appraisal Checklist

| **Title** | **1. Was the sample representative of the target population?** | **2. Were study participants recruited in an appropriate way?** | **3. Was the sample size adequate?** | **4. Were the study subjects and the setting described in detail?** | **5. Was the data analysis conducted with sufficient coverage of the identified sample?** | **6. Were objective, standard criteria used for the measurement of the condtion?** | **7. Was the condition measured reliably?** | **8. Was there appropriate statistical analysis?** | **9. Are all important confounding factors/subgroups/differences identified and accounted for? (are covariates examined)** | **10. Were subpopulations identified using objective criteria?** | **Evaluation of risk of bias (Low; Medium; High; High risk and exclude)** |
| --- | --- | --- | --- | --- | --- | --- | --- | --- | --- | --- | --- |
| *Adegunloye et al. 2010 | Yes | Yes | Yes | Yes | Yes | Yes | Yes | Yes | No | Yes | Low |
| *Ajiboye et al. 2009 (same sample as Adegunloye) | Yes | Yes | Yes | Yes | Yes | Yes | Yes | Yes | No | Yes | Low |
| *Yusuf et al. 2011 (same sample as Adegunloye) | Yes | Yes | Yes | Yes | Yes | Yes | Yes | Yes | No | Yes | Low |
| *Atilola et al. 2017 "Correlations..." | Yes | Yes | Yes | Yes | Yes | Yes | Yes | Yes | No | Yes | Low |
| *Atilola et al. 2017 "Status..." | Yes | Yes | Yes | Yes | Yes | Yes | Yes | Yes | No | Yes | Low |
| *Beyen et al. 2017 | Yes | Yes | Yes | Yes | Yes | Yes | Yes | Yes | No | Yes | Low |
| *Dachew et al. 2015 (same sample as Beyen) | Yes | Yes | Yes | Yes | Yes | Yes | Yes | Yes | No | Yes | Low |
| *Dadi et al. 2016[94] (same sample as Beyen) | Yes | Yes | Yes | Yes | Yes | Yes | Yes | Yes | No | Yes | Low |
| Abdulmalik et al. 2014 | Yes | Yes | Yes | Yes | Yes | Yes | Yes | Yes | No | Yes | Low |
| Agbahowe et al. 1998 | Yes | Yes | Yes | Yes | Yes | Yes | Yes | Yes | No | Yes | Low |
| Agboola et al. 2017 | Yes | Yes | Yes | Yes | Yes | Yes | Yes | Yes | No | Yes | Low |
| Atilola et al. 2014 | Yes | Yes | Yes | Yes | Yes | Yes | Yes | Yes | No | Yes | Low |
| Atilola et al. 2016 | Yes | Yes | Yes | Yes | Yes | Yes | Yes | Yes | Unclear | Yes | Low |
| du Plessis et al. 2017 | Yes | Yes | Yes | No | Yes | Unclear | Unclear | Yes | No | Yes | Low |
| Fatoye et al. 2006 | Maybe | Yes | Yes | Yes | Yes | Yes | Yes | Yes | No | Yes | Low |
| Ibrahim et al. 2015 | Yes | Yes | Unclear | Yes | Yes | Yes | Yes | Yes | No | Yes | Low |
| Majekodunmi et al. 2017 | Yes | Yes | Yes | Yes | Yes | Yes | Yes | Yes | No | Yes | Low |
| Mela et al. 2014 | Yes | Yes | Yes | Yes | Yes | Yes | Yes | Yes | No | Yes | Low |
| Nseluke & Siziya 2011 | Yes | Yes | Yes | No | Yes | Yes | Unclear | Yes | No | Yes | Low |
| *Olashore et al. 2016 | Yes | Yes | Yes | Yes | Yes | Yes | Yes | Yes | No | Yes | Low |
| *Olashore et al. 2017 | Yes | Yes | Yes | Yes | Yes | Yes | Yes | Yes | No | Yes | Low |
| Osasona & Koleoso 2015 | Yes | Yes | Yes | Yes | Yes | Yes | Yes | Yes | Unclear | Yes | Low |
| Schaal et al. 2012 | Yes | Yes | Yes | Yes | Yes | Yes | Yes | Yes | Yes | Yes | Low |
| *Uche & Princewill 2015 | Yes | Yes | Yes | Yes | Yes | Yes | Yes | Yes | Yes | Yes | Low |
| *Uche & Princewill 2016 | Yes | Yes | Yes | Yes | Yes | Yes | Yes | Yes | No | Yes | Low |
| *Armiya'u et al. 2013 " Prevalence of..." | Yes | Yes | Yes | Yes | Yes | Yes | Unclear | Yes | No | Yes | Medium |
| *Armiya'u et al. 2013 "A study of..." | Yes | Unclear | Yes | No | Yes | Yes | Yes | Yes | No | Yes | Medium |
| Akkinawo 1993 | Yes | Yes | Yes | No | Yes | Yes | Yes | Yes | No | Yes | Medium |
| Atilola 2012 "Different points..." | Yes | Yes | Yes | Yes | Yes | Yes | Yes | Yes | No | Yes | Medium |
| Atilola 2012 "Prevalence and correlates..." | Yes | Yes | Yes | Yes | Yes | Yes | Yes | Yes | No | Yes | Medium |
| Barrett et al. 2007 | Yes | Yes | Yes | No | Yes | Unclear | Unclear | Yes | Yes | Yes | Medium |
| Bella et al. 2010 | Yes | Unclear | Unclear | Yes | Yes | Yes | Yes | Yes | No | Yes | Medium |
| Calitz et al. 2006 | Yes | Yes | Yes | No | Yes | Unclear | Unclear | Yes | No | Yes | Medium |
| Hayward et al. 2010 | Yes | Yes | Yes | No | Yes | Unclear | Unclear | Yes | Unclear | Yes | Medium |
| *Issa et al. 2009 (same sample as Adegunloye) | Yes | Yes | Yes | Yes | Unclear | Yes | Yes | Yes | No | Yes | Medium |
| Kanyanya 2007 | No | Unclear | Unclear | Yes | Yes | Yes | Yes | Yes | No | Yes | Medium |
| Khoele et al. 2016 | Yes | Yes | Unclear | Yes | Yes | Unclear | Unclear | Yes | No | Yes | Medium |
| Marais & Subramaney 2015 | Unclear | Yes | Yes | Yes | Yes | Unclear | Unclear | Yes | No | Yes | Medium |
| Matete 1991 | Yes | Yes | Unclear | Yes | Yes | Unclear | Unclear | Yes | No | Yes | Medium |
| Menezes 2010 | Unclear | Yes | Unclear | Yes | Yes | Yes | Unclear | Yes | No | Yes | Medium |
| Menezes et al. 2007 | Yes | Yes | Yes | Yes | Yes | Yes | Yes | Yes | No | Yes | Medium |
| Naidoo & Mkize 2012 | Yes | Yes | Yes | Yes | Yes | Yes | Yes | Yes | No | Yes | Medium |
| Odejide 1981 | Unclear | Yes | No | Yes | Yes | Yes | Unclear | Yes | No | Yes | Medium |
| Offen et al. 1986 | Unclear | Unclear | Yes | No | Yes | Unclear | Unclear | Yes | No | Yes | Medium |
| Prinsloo & Hesselink 2014 | Yes | Yes | Yes | No | Yes | Unclear | Unclear | Yes | No | Yes | Medium |
| Strydom et al. 2011 | Yes | Yes | Yes | Yes | Unclear | Unclear | Unclear | Yes | No | Yes | Medium |
| Touari et al. 1993 | Yes | Yes | Yes | No | Yes | Unclear | Unclear | Yes | No | Yes | Medium |
| Turkson & Asante 1997 | Yes | Yes | Yes | No | Yes | Unclear | Unclear | Yes | No | Yes | Medium |
| Verster & Van Rensburg 1999 | Yes | Yes | Yes | Yes | Yes | Unclear | Yes | Yes | Yes | Yes | Medium |
| Yusuf & Nuhu 2009 | Yes | Yes | Unclear | No | Yes | Unclear | Unclear | Yes | No | Yes | Medium |
| Ogunlesi et al. 1988 | Yes | Yes | Yes | Yes | Yes | Unclear | Unclear | Yes | No | Yes | Medium |
| Zabow 1989 | Yes | Yes | Yes | No | Yes | Unclear | Unclear | Yes | No | Yes | Medium |
| *Mafullul 2000 | Yes | Yes | Yes | No | Yes | Unclear | Unclear | Yes | No | Yes | High |
| *Mafullul et al. 2001 | Yes | Yes | Yes | No | Yes | Unclear | Unclear | Yes | No | Yes | High |
| Buchan 1976 | No | Yes | Yes | No | Yes | No | No | Yes | No | No | High |
| Hemphill & Fisher 1980 | Yes | Yes | Yes | No | Unclear | Unclear | Unclear | Yes | No | Yes | High |
| Mbassa 2009 | No | No | No | Yes | Yes | Yes | Unclear | No | No | No | High |
| Muluka & Acuda 1978 | Yes | Yes | Unclear | No | Yes | Unclear | No | Yes | No | Yes | High and exclude |

**S6. Robustness check – Sub-grouping by institution and by data collection method**

*(only Barrett et al., 2007 is different between the sub-groups as it is conducted in a forensic ward and uses an instrument, while all other forensic ward studies use clinical records and all prison studies use instruments)*

|  | **Mood disorder** | | **Mental ill health** | | **Substance use** | | **Psychotic disorders** | |
| --- | --- | --- | --- | --- | --- | --- | --- | --- |
|  | **Data** | **Inst** | **Data** | **Inst** | **Data** | **Inst** | **Data** | **Inst** |
| Pooled prevalence (95% CI) | CR: 0.06  (0.03-0.08)  Tool: 0.31 (0.19-0.44) | For: 0.06  (0.03-0.08)  Pri: 0.33  (0.20-0.46) | CR: 0.57  (0.42-0.73)  Tool: 0.60  (0.47-0.72) | For: 0.57  (0.42-0.73)  Pri: 0.60  (0.47-0.72) | CR: 0.40  (0.24-0.56)  Tool: 0.32  (0.22-0.41) | For: 0.39  (0.22-0.56)  Pri: 0.35  (0.25-0.44) | CR: 0.42  (0.32-0.52)  Tool: 0.05  (0.02-0.07) | For: 0.44  (0.34-0.54)  Pri: 0.01  (0.00-0.02) |

Data = sub-grouped by data collection method, Inst = sub-grouped by institution type

CR = clinical record, Tool = diagnostic or screening tool, For = forensic institution, Pri = prison

**S7. Census sampling only**

*This sensitivity analysis was conducted to examine heterogeneity based on sampling technique. Here, we used only studies that used census sampling as that is the most homogenous type of sampling; randomization can vary substantially based on the method used to randomize, and the randomization method was not stated in most of the included studies*

|  | **Mental ill health** | **Mood disorder** | **Substance use** | **Psychotic disorders** |
| --- | --- | --- | --- | --- |
| Pooled prevalence (95% CI) | 0.57 (0.41-0.73) | 0.13 (0.07-0.20)* | 0.38 (0.22-0.54) | 0.37 (0.31-0.44) |
| Heterogeneity chi^2^ | 1454.38 | 1616.28 | 856.87 | 2927.57 |
| I^2 *^ | 99.24% | 99.26% | 98.83% | 99.28% |
| Number of studies | 12 | 13 | 11 | 22 |

**Only mood disorders had a substantially different point prevalence between this analysis and overall adult prevalence: 22% of mood disorders (95% CI: 16%-28% among adults overall, Figure 3). We hypothesize that this may be a combination of the fact that fewer of the mood disorder studies used census sampling than the other disease categories and that most studies using census sampling were conducted in forensic wards, which had a lower prevalence of mood disorders (there was substantial confounding present).*

**S8. Additional Details of Intervention Studies**

| **Reference** | **Description of intervention** | **Control or comparison condition** | **Length of intervention** | **Method for assessment of intervention** | **Sample size** | **Selection of sample** | **Outcomes tested** | **Results of intervention** |
| --- | --- | --- | --- | --- | --- | --- | --- | --- |
| Eseadi et al. 2017 | Participants in the treatment group took part in the CBC program based on the REBT depression manual for 12 sessions (sessions were held twice per week) over 6 consecutive weeks. Sessions lasted 50 min each. | Participants in the control group took part in conventional counseling (not anchored on any theory) for 12 sessions.Sessions lasting 50 min each were held twice per week for 6 consecutive weeks. | 6 weeks | BDI measure before/after. A pretest (Time 1) for depression using the BDI was given to all participants in both groups before the intervention to assess participants’ level of depression. After the intervention, a posttest (Time 2) for depression was given after 3 months from completing the study to all participants in both groups using the same measure. ANOVA was used to explore the effect of group-focused CBC on depressive symptoms in the treatment group compared with the control group, and Mann–WhitneyUTest. | 30 (15 in each group) | census, screening for BDI >29, then randomly allocated to treatment/control | BDI score | ANOVA significant treatment by time interaction effect for cognitive behavioral coaching program on depression as measured by BDI: decrease from pre to post-test BDI score (p = 0.000; *F*(1, 28) = 3.995; η2p =.998) for the CBC group compared to control. Mann–WhitneyUTest: significant decreases from Time 1 to Time 2 on depression (*T* = -7.707; p = .000) for the CBC Group. |
| Onyechi et al. 2017 | Group-focused cognitive behavioral health education training developed by the researchers. Training had 5 modules (1. cognitive restructuring; 2. mindfulness training; 3. self-modification training; 4. impulsive tolerance and emotional regulation training; 5. psychodrama). Every treatment module lasted for 2 weeks with sessions of 40 minutes each. Treatment process took up to 20 sessions and was scheduled for twice a week. Treatment was a total of 10 weeks plus 4 weekly follow-up sessions held after 6 months. Each session lasted for 40 minutes. | Control group participants were given 10 weeks of conventional counseling. | 10 weeks treatment plus 4 weekly follow-up sessions held after 6 months. | CDS-12 score. ANOVA of pre-post treatment | 20 (10 treatment, 10 control). | census, screening on CDS | CDS-12 score | After the cognitive behavioral intervention, prisoners in the treatment group has significantly lower post-intervention CDS-12 scores than the control group's post-intervention scores (p = 0.00; F(1,18)=4.177; η2p = 0.99). |
| Martyns-Yellowe 1993 | One group recieved Flupenthixol decanoate (40mg per injection), Each subject received a total of eight injections: 4 fortnightly injections, followed by 3 weekly injections, and by two further monthly injections. | Comparison group received Clopenthixol decanoate (200mg per injection), following the same injection scheme as Flupenthixol group. | 24 weeks | Baseline (week 0) and final (week 24) BPRS assessments. 3 parallel monthly clinical progress assessments made on consensus basis at ward rounds. Weekly side-effects assessment using checklist by psychiatric nurses. Final assessments made 4 weeks after 8th injection at 24th week. Assessed with t-tests between groups comparing the change in symptoms from baseline to the end of treatment. | 36 (18 in each group) | census | BPRS scores and side effects | 57.1% drop in BPRS symptoms in the Flupenthixol group (t= 4.19; df=15; p < 0.001) and 43.4% drop in the Clopenthixol group (t=3.16; df=15; p < 0.01). Flupenthixol group had better symptom reducation respsone than the Clopenthixol group (t = 3.35; df=15; p < 0.01). |

**S9. Descriptions of institutions, health system, or policies**

| **Reference** | Atilola et al. 2017 "Status..." | Dube-Mawerewere 2015 | Fatoye et al. 2006 | Gaum et al. 2006 | Hayward et al. 2010 | Kaliski et al. 1997 | Kidia, et al. 2017 | Mafullul 2000 | Naidoo & Mkize 2012 | Osasona & Koleoso 2015 | Sukeri et al. 2016 | Topp et al. 2016 | Zabow 1989 |
| --- | --- | --- | --- | --- | --- | --- | --- | --- | --- | --- | --- | --- | --- |
| **Study design** | Prevalence | Structured health system review | Prevalence | Qualitative | Prevalence | Qualitative | Structured health system review | Prevalence | Prevalence | Prevalence | Prevalence | Qualitative | Prevalence |
| **Study Setting** | Youth institution | Health system | Prison | Prison | Forensic ward | Prison | Health system | Prison | Prison | Prison | Forensic ward | Prison | Forensic ward |
| **Facilities Included** | 5 youth correctional institutions in Lagos, Nigeria (2 female, 3 male institutions) | All forensic psychiatry settings in Zimbabwe | Medium security prison in Ilesa, Nigeria | Pollsmoor Prison, South Africa | Zomba Mental Hospital, Malawi | Valkenberg Hospital, South Africa | All forensic psychiatry settings in Zimbabwe | Jos Federal Prison, Nigeria | Westville Correctional Centre, South Africa | Medium security prison in Benin City, Nigeria | Forensic mental hospital: Fort England Hospital, South Africa | 4 prisons, Zambia | Forensic Psychiatry Unit at Valkenberg Hospital, South Africa |
| **How was data on conditions collected?** | Questionnaire, interviews with all operational on staff on site at time of study interviewed | Purposefully and theoretically sampled 32 participants in semi-structured interviews. Sample included the judicial team, the medical team, forensic psychiatric patients, and relatives of forensic psychiatric patients. | Questionnaire | 3 focus group interviews with 6 participants in prison each who are active therapeutic clients and also recidivists (18 males), and semi-structured interviews with first 10 participants (5 males, 5 females) who voluntarily agreed to participate, had no previous convictions, and had spent time in prison at least once prior to their current sentence, but on a different charge. Other stakeholders interviewed: the judicial team, the medical team, forensic psychiatric patients, and relatives of forensic psychiatric patients. | Secondary records | Interview and secondary records | Emerald national-level needs assessment methods. Qualitative interviews with a range of key stakeholders in health and mental health using Emerald interview guide for national-level policy makers. Included policy makers, administrators, providers, and researchers, but not service users. Used Emerald policy checklists for analysis of mental health laws and policies and publicly available data about mental health. | Secondary records | Questionnaire | Questionnaire | Questionnaire designed to collect data on the number of observations conducted, state patients admitted, average length of stay, and staff. Calculated psychiatric service needs for a provincial prison mental health service using pubic records. | Interviews of a clustered random sample of 79 male individuals across four prisons purposively selected, as well as 32 prison officers, policy makers and health care workers. Largely in- ductive thematic analysis was guided by the concepts of dynamic interaction and emergent behavior. ***excluded those with a known history of mental illness | Secondary records |
| **Institutional Conditions:** | | | | | | | | | | | | | |
| Insufficient physical resources (living conditions, clothes, etc.) | x |  | x | x |  |  | x |  |  | x |  | x |  |
| Insufficient food |  |  | x |  |  |  | x |  |  | x |  | x |  |
| Insufficient psychiatric medicines |  |  | x |  |  |  | x |  |  |  |  | x |  |
| Insufficient of human resources for health | x | x |  | x | x |  | x | x | x | x | x | x |  |
| Lack of psychosocial services | x |  |  | x | x |  | x | x | x | x | x |  |  |
| Long waiting times for trial or release/slow bureaucratic systems | x |  |  | x |  |  | x | x |  |  |  |  |  |
| Conditions have negative impact on mental health |  | x |  | x |  |  |  |  |  |  |  | x |  |
| Limited rehabilitation, recreational, or vocational services | x | x |  | x |  |  | x |  |  |  |  |  |  |
| **Policy / Law:** | | | | | | | | | | | | | |
| Laws or policies exist to help detained people but are not implemented |  |  |  | x |  | x | x |  |  |  |  |  |  |
| Lack of timely psychiatric assessment | x |  |  | x |  |  | x | x |  |  | x |  |  |
| Lack of communication between medical and justice systems |  |  |  |  |  | x | x |  |  |  | x |  |  |
| Many people who go between mental health services and criminal justice system |  | x |  |  | x |  |  |  |  |  |  |  |  |
| Lack of rehabilitation or community re-integration plans | x | x |  | x |  |  | x | x |  |  |  |  |  |

**S10. Reported Associated Factors**

Association tests conducted to explore factors associated with a psychiatric outcome.

| **Reference**  *If same sample as another study in list | **Reported Associated Factors:** Association tests conducted to explore factors associated with a psychiatric outcome. Includes results of any test (univariate and multivariate regression, t-test, chi square test, etc.).  *Indicates significant relationship (p < 0.05). No “*” indicates lack of significance. |
| --- | --- |
| Abdulmalik et al. 2014 | frequency of visits*; prison term length; number of previous arrests; position in cell; age; level of education; religion; employment status; income; marital status |
| *Adegunloye et al. 2010 | age*; parents living apart*; duration of prison stays; source of referral; ethnicity; religion; birth order |
| *Yusuf et al. 2011 (same sample as Adegunloye) | age*; religion*; parents living status*; birth order*; prison term length (duration of time spent); source of referral |
| Agbahowe et al. 1998 | participant’s view of others in prison*; current medical complaints*; previous psychiatric history*; type of sentence; length of stay prison; length of sentence; forensic history; charge; type of imprisonment (detainees, convicted, or condemned); awareness of offense; mental illness at time of offense; physical health or chronic medical/surgical illness; age |
| Akkinawo 1993 | prison term length*; age*; marital status* |
| *Armiya'u et al. 2013a | co-morbid physical illness*; prison term length |
| *Armiya'u et al. 2013b | trial status (awaiting trial or convicted)*; age* |
| Atilola 2012b "Prevalence and correlates..." | school status at point of contact with juvenile justice (not in school vs in school)*; current caregiver*; early childhood caregiver*; number of different people participant had lived with during lifetime*; birth order* |
| Atilola et al. 2014 | mean incident of traumatic event*; nature of traumatic event*; |
| Atilola et al. 2016 | type of detention (young offender)*; age*; gender*; length of staying on the streets or by self*; truancy*; ever registered to learnt a trade*; school status as at point of contact with juvenile justice; self-reported preexisting school-related problems before admission into institution |
| Atilola et al. 2017a "Correlations..." | quality of life*; gender; reason for detention |
| Bella et al. 2010 | comorbid psychiatric variables* |
| *Beyen et al. 2017 | imprisonment status (lifetime prisoner vs not lifetime)*; satisfaction with day to day activity before imprisonment*; thinking impossibility not to run the life they had before*; discrimination due to crime*; acceptance of crime*; place of the prison*; thinking to commit suicide*; having plan to commit suicide*; previous psychiatric history*; marital status*; family members with mental illness*; social support* |
| *Dachew et al. 2015 (same sample as Beyen) | long duration of stay in the prison*; low to no satisfaction with prison services *; place of prison* |
| *Dadi et al. 2016 (same sample as Beyen) | feeling unhappy with life before imprisonment*; place of the prison*; smokers*; discrimination due to crime; acceptance of crime |
| Calitz et al. 2006 | charge*; violent crime* |
| du Plessis et al. 2017 | not accountable for charge* |
| Fatoye et al. 2006 | imprisonment status*; self-reported mental health*; self-reported prison accommodation rating*; self-reported prison healthcare rating*; prison term length; forensic history; charge; self-reported prison feeding rating; visits by family/friends; previous psychiatric history; age; gender; education; religion; marital status; current caregiver; financial support |
| Hayward et al. 2010 | charge*; prison term length |
| Idemudia 1998 | prison term length* |
| Idemudia 2007 | charge (nature of crimes)* |
| Ineme & Osinowo 2016 | comorbid psychiatric variable (self-harm urges)*; prior substance use*; female gender* |
| Issa et al. 2009 | prison term length; source of referral for institution; age; ethnicity; religion; parents living together; birth order |
| Kaliski et al. 1997 | able to distinguish between a guilty/not guilty plea*; forensic history; acceptance of crime; awareness of court procedure |
| Liddicoat et al. 1972 | charge*; personality traits* |
| Majekodunmi et al. 2017 | presence of physical complaints* (significant among awaiting trial participants only, not convicted participants); chronic illness* (significant among awaiting trial participants only); family history of psychiatric illness* (significant among convicted participants only); age; religion; marital status; employment; educational status; trial status; type of offense |
| Mela, M., et al. 2014 | charge (juvenile offense)*; comorbid psychiatric variable*; previous psychiatric history*; history of abuse* |
| Naidoo & Mkize 2012 | trial status (awaiting trial or sentenced)* |
| Nseluke & Siziya 2011 | marital status*; trial status; age; gender; education; religion; employment status; income |
| *Olashore et al. 2016 | forensic history (previous detention)*; parents' marital status*; number of siblings*; family setting*; who brought the participant up*; age at parental separation/divorce/death*; duration of prison stay; reason for detention; age; ethnicity; religion; frequency of religious participation |
| *Olashore et al. 2017 | reason for detention (juvenile offender status)* |
| Osasona & Koleoso 2015 | trial status (remand/awaiting trial or various sentences)*; self-reported prison accommodation rating*; self-reported prison feeding rating*; self-reported prison healthcare rating*; self-reported mental health*; previous psychiatric history*; self-reported physical health*; age*; marital status*; prison term length; forensic history; gender; education; religion; employment status; family type; social support; |
| Prinsloo & Hesselink 2014 | charge* |
| Prinsloo & Ladikos 2007 | forensic history*; being segregated due to history of maladjustment, disciplinary problems and other institutional infractions*; anger* |
| Prinsloo 2013 | prison term length*; criminal tendencies*; anti-social personality variables*; conduct problems*; lack of remorse and empathy with victims*; not engaging in criminal behavior for the sake of excitement*; less prone to use dangerous weapons*; substance abuse immediately prior or during a crime*; anger* |
| Schaal et al. 2012 | participation in killing*; reconciliation score*; number of traumatic events*; age |
| Stephens et al. 2006 | lifetime occurrence of sexually transmitted infection* |
| Touari et al. 1993 | charge (type of crime)*; comorbid psychiatric variable*; previous psychiatry history*; mandatory vs optional psychiatric evaluation*; age*; occupation before arrest*; family size*; familial status (single or other)*; raised by 2 parents*; forensic history; attended school |
| *Uche & Princewill 2016 | previous psychiatric history*; smoking habit; other psychoactive substance use; medical or surgical condition; disabilities or limitation; on medication; past medical history; physical status; HIV status; history of sexual activity before detention |
| *Uche & Princewill 2015 | place of living*; age*; marital status*; living arrangements (people lived with before prison)*; ethnicity; religion; employment status; social class; occupation before arrest |
| Weierstall et al. 2011 | prison term length (duration of time spent)*; appetitive aggression score*; witnessed more violent events*; committed more types of crimes*; place of the prison*; male gender*; trial status |
| Yusuf & Nuhu 2009 | charge (homicide)*; offending behavior* |

**S11. Secondary Outcomes**

Outcomes reported other than forensic or psychiatric outcomes

| **Reference**  *If same sample as another study in list | **Secondary outcomes reported other than forensic or psychiatric outcomes** |
| --- | --- |
| Abdulmalik et al. 2014 | institution conditions; justice system qualities |
| Agbahowe et al. 1998 | physical illness; institution conditions (rating) |
| Agboola et al. 2017 | physical illness |
| Armiya'u et al. 2013a | physical illness |
| Atilola et al. 2017b "Status..." | institution conditions |
| *Beyen et al. 2017 | satisfaction with the care given in prison |
| *Dachew et al. 2015  (same sample as Beyen) | satisfaction with the care given in prison |
| *Dadi et al. 2016  (same sample as Beyen) | satisfaction with the care given in prison |
| Fatoye et al. 2006 | institution conditions (rating) |
| Hayward et al. 2010 | institution conditions; justice system qualities |
| Idemudia 1998 | stress |
| Kanyanya 2007 | sexual history; history of assault |
| Mafullul 2000 | institution conditions; justice system qualities |
| Majekodunmi et al. 2017 | physical illness; physical complaints |
| Marais & Subramaney 2015 | 3-year follow-up of living status (detention; living in community; method of departure) |
| Naidoo & Mkize 2012 | physical illness; institution conditions |
| Osasona & Koleoso 2015 | physical health; institution conditions (rating) |
| Stephens et al. 2006 | physical illness (sexually transmitted infection) |
| Uche & Princewill 2016 | physical illness; history of sexual activity before detention |
| Weierstall et al. 2011 | appetitive aggression |
| Zabow 1989 | institution conditions; justice system qualities |
